# *In vitro* and *in vivo* evaluation of immortalized hepatocyte encapsulated click-microbeads with RGD peptide for treatment of liver failure in male rats

**DOI:** 10.3389/fbioe.2025.1629228

**Published:** 2025-07-15

**Authors:** Su Yee Win, Pinunta Nittayacharn, Jatupoom Ngernmark, Mongkol Chavalitsarot, Chitinart Thedrattanawong, Khanit Sa-ngiamsuntorn, Suradej Hongeng, Norased Nasongkla

**Affiliations:** ^1^ Department of Biomedical Engineering, Faculty of Engineering, Mahidol University, Nakhon Pathom, Thailand; ^2^ Department of Biochemistry, Faculty of Pharmacy, Mahidol University, Bangkok, Thailand; ^3^ Department of Pediatrics, Faculty of Medicine, Ramathibodi Hospital, Mahidol University, Bangkok, Thailand

**Keywords:** cell encapsulation, immortalized hepatocytes, click-RGD microbeads, acute liver failure, *in vivo*

## Abstract

Cell encapsulation in biocompatible microbeads offers a promising strategy for cell-based therapy in acute liver failure (ALF). This study evaluates the use of immortalized hepatocyte cells (imHCs) encapsulated in click-arginyl glycyl aspartic acid (click-RGD)-modified alginate microbeads, focusing on their biocompatibility and therapeutic potential. *In vitro* assessments showed that click-RGD microbeads significantly enhanced cell viability on day 4, spatial distribution, and hepatocyte function, evidenced by increased albumin on day 14 and alpha-fetoprotein (AFP) secretion compared to unmodified alginate microbeads. For *in vivo* testing, ALF was induced in Sprague-Dawley male rats using D-galactosamine (D-gal), followed by intraperitoneal administration of imHCs-loaded click-RGD microbeads in the treated group and CMRL medium injection in the control group. Treated rats exhibited faster reductions in aspartate aminotransferase (AST) and alanine aminotransferase (ALT) levels, higher albumin production, and improved liver histology, characterized by reduced necrosis and the absence of inflammation, on day 14 after treatment. No adverse host responses were observed, confirming the biocompatibility of the microbeads. These findings support the potential of click-RGD microbeads as a therapeutic platform for ALF, warranting further studies on long-term implantation, immune response, and co-encapsulation strategies.

## Graphical Abstract

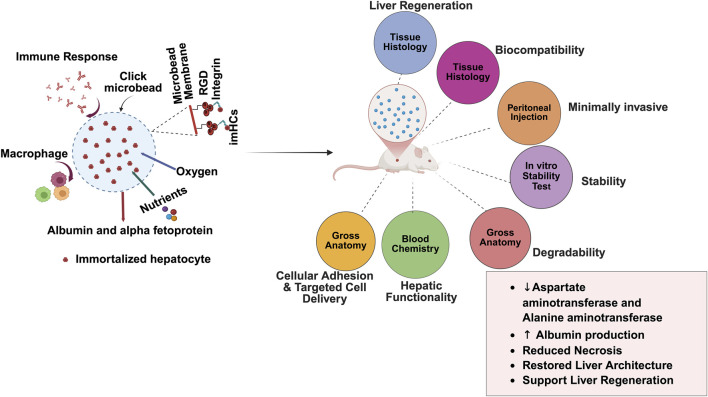

## 1 Introduction

The liver exhibits remarkable regenerative capacity, with rodents capable of restoring up to two-thirds of their hepatic mass within 7–10 days post-resection. In humans, liver mass restoration after hepatectomy typically takes about 3 months (Yagi et al., 2020). Considering the liver’s notable regenerative capacity, there has been increasing interest in strategies, such as cell-based therapies, to enhance its healing process for critical conditions in liver failure (Bhatt et al., 2024). However, the biocompatibility of the biomaterials used in cell-based therapies is essential for tissue engineering and liver regeneration. Cell-based therapies also present promising solutions to address organ shortages, mitigate surgical complications, and reduce the need for lifelong immunosuppression (Zhang et al., 2021). Furthermore, encapsulating hepatocytes within a biocompatible, semipermeable membrane offers an immunoprotective barrier, thereby maintaining hepatocyte viability and functionality (Somo et al., 2018). This membrane permits the bidirectional diffusion of essential molecules, including oxygen, nutrients, and metabolic waste, while restricting the passage of immune components such as antibodies and immune cells (Orive et al., 2003). This strategy not only obviates the need for immunosuppressive treatments but also facilitates essential cellular exchanges with the host environment (Saimok et al., 2023).

Among various cell encapsulation technologies, alginate-based microbeads are extensively employed. However, their long-term applicability in transplantation is limited by poor degradability, suboptimal biocompatibility, and insufficient mechanical strength. These challenges highlight the need for advanced biomaterials to ensure sustained hepatocyte function *in vivo*. RGD peptides offer a promising strategy to address these issues.

**FIGURE 1:**
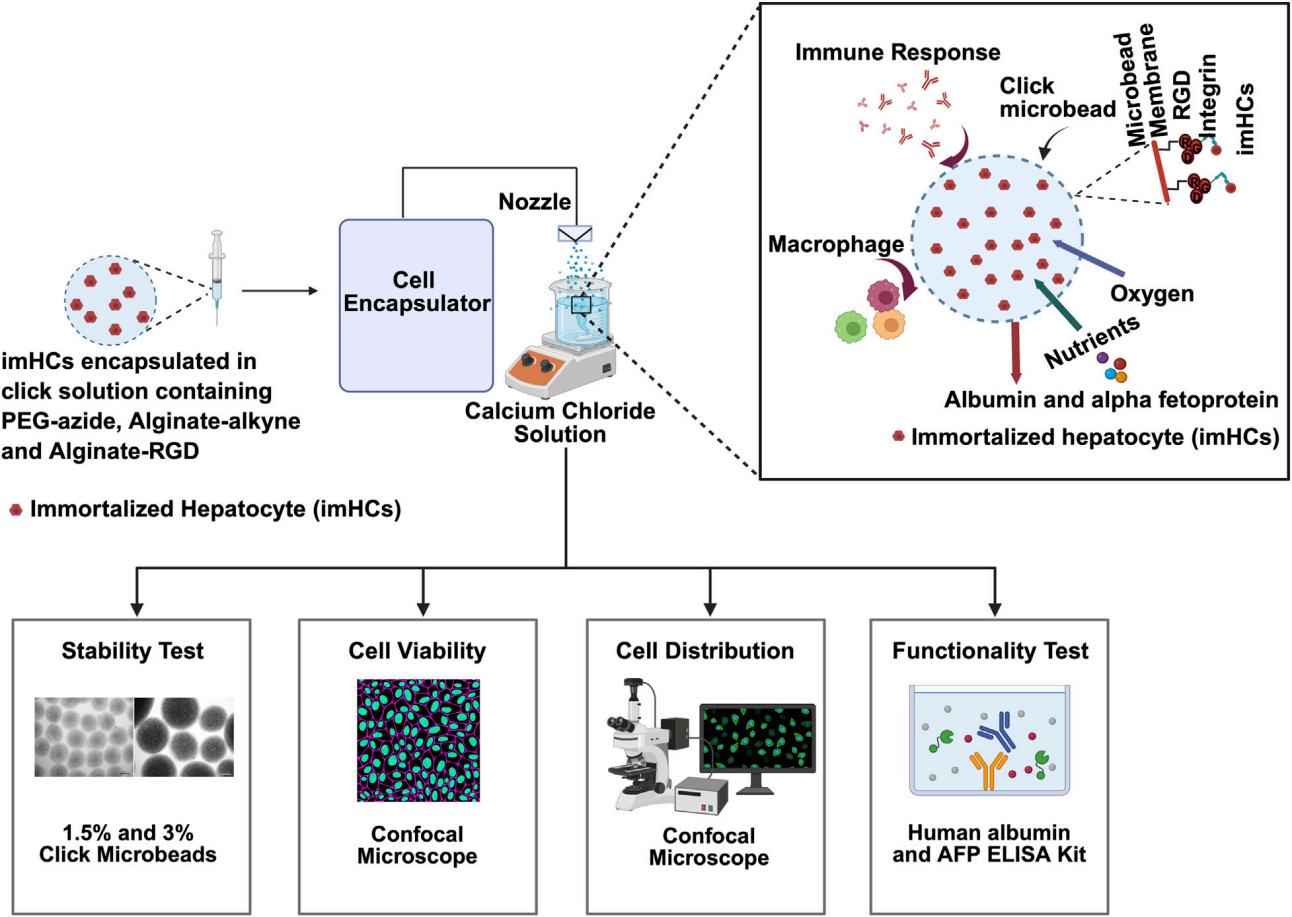
Schematic overview of the encapsulation and evaluation process for immortalized hepatocytes (imHCs) within click-crosslinked microbeads for liver regenerative therapy. imHCs were encapsulated in a bio-orthogonal click-crosslinking solution composed of PEG-azide, alginate-alkyne, and alginate-RGD using a cell encapsulator system. The microbeads were formed via extrusion through a nozzle into a calcium chloride bath, creating uniformly sized constructs for *in vitro* and *in vivo* testing. The inset (top right) illustrates the microenvironment within a click-RGD microbead, showing imHCs supported by an RGD-modified alginate matrix that facilitates integrin binding, allowing for nutrient and oxygen exchange, albumin and alpha-fetoprotein (AFP) secretion, and minimized immune activation due to the biocompatible membrane (created with Biorender.com).

Building on these advancements, click microbeads functionalized with RGD provide significant advantages in encapsulating cells while maintaining their structure and function. Encapsulating imHCs in these microbeads enhances cell survival and interactions (Abbas et al., 2025). In this study, click chemistry ensures precise functionalization and crosslinking, thereby creating a stable environment for cell growth and differentiation. Moreover, this study evaluates the biocompatibility of click-RGD microbeads encapsulating imHCs and their potential application in liver regeneration therapies. In Figure 1, bifunctional click-RGD microbeads were synthesized using copper-catalyzed azide-alkyne cycloaddition (CuAAC) reaction, combining polyethylene glycol-azide, alginate-alkyne, and alginate-RGD to form stable 1,2,3-triazole bonds. These microbeads enhanced hydrophilicity, mechanical strength, and cellular adhesion. Firstly, *in vitro* assessments demonstrated improved hepatocyte survival, albumin secretion, and AFP secretion, confirming the enhanced viability and performance of the modified microbeads. *In vivo*, imHCs were encapsulated in click-RGD microbeads for hepatic regeneration and injected peritoneally into an ALF rat model. Various physiological, biochemical parameters, and histological examinations were assessed to evaluate the therapeutic efficacy of imHCs encapsulated in click-RGD microbeads. This study provides insights into the potential of imHCs-encapsulated click-RGD microbeads for liver regeneration after acute liver failure.

## 2 Materials and Methods

### 2.1 Materials

D-galactosamine (Atomax Chemicals, China), sodium alginate (BUCHI, Switzerland), and calcium chloride (Ajax Finechem, Australia) were used. Poly (ethylene glycol) 6 kDa (Sigma-Aldrich, Germany), tosyl chloride, sodium azide, and sodium ascorbate (Tokyo Chemical Industry, Japan) were purchased for synthesis. RGD peptide (Biomatik, United States) and sodium hydroxide, tetrahydrofuran, diethyl ether, dichloromethane, dimethylformamide, N-Hydroxysuccinimide (NHS), 1-Ethyl-3-(3-dimethylaminopropyl)carbodiimide (EDC), propargylamine, and HEPES (Sigma-Aldrich, United States), Hoechst 33342 and Propidium iodide (PI) (Sigma-Aldrich, St. Louis, MO, United States) were purchased. Cell culture media, including DMEM/F12, Fetal Bovine Serum (FBS), GlutaMAX, PBS, penicillin-streptomycin, and TrypLE™ Express Enzyme (Gibco, United States), were used. CMRL-1066 medium (initially developed by Connaught Medical Research Laboratories) (Pan Biotech GmbH, Germany) was used. imHCs were provided by Assistant Professor Khanit Sa-ngiamsuntorn (Mahidol University, Thailand; Ethical approval: MUSC66-048-678).

### 2.2 Synthesis of click components

To encapsulate imHCs within click-RGD microbeads, three core components were synthesized: PEG-azide, alginate-alkyne, and alginate-RGD conjugates. The encapsulation matrix was constructed using copper(I)-catalyzed azide-alkyne cycloaddition (CuAAC), a widely employed bioorthogonal click reaction known for its efficiency and biocompatibility. These synthesized conjugates served as the primary building blocks for the formation of microbeads. Detailed procedures for the chemical reaction of each component are provided in the supporting information in Supplementary Figure S1.

#### 2.2.1 PEG-azide synthesis

PEG-azide was synthesized through a two-step procedure. In the first step, hydroxyl-terminated polyethylene glycol (PEG) (30 g) was dissolved in dichloromethane, and triethylamine and tosyl chloride were added. The reaction mixture was stirred at 0°C for 36 h in an ice bath. The solvent was removed by rotary evaporation, yielding PEG-tosyl as a solid powder. In the second step, PEG-tosyl (22 g) was dissolved in ethanol at 50°C, and sodium azide (NaN_3_) was added. The reaction was refluxed at 85°C for 16 h. After completing the reaction, the ethanol was evaporated, and acetone was added to remove unreacted sodium azide. The mixture was centrifuged at 3,000 rpm for 10 min to isolate PEG-azide (Edward Semple et al., 2016). Experimental details are in the supporting information in Supplementary Figure S2.

#### 2.2.2 Alginate-alkyne and alginate-RGD synthesis

Alginate-alkyne and alginate-RGD conjugates were synthesized by activating alginate’s carboxyl groups using NHS and EDC. This activation enabled the conjugation of alkyne and RGD to the alginate backbone. The alkyne conjugation was achieved via amide bond formation with propargylamine, using EDC and NHS to activate the carboxyl groups and form an NHS-ester intermediate (Jain et al., 2021; Sandvig et al., 2015). RGD conjugation was carried out similarly. For the conjugates, 10 mL of a 1% alginate solution was mixed with 147 mg of NHS and 500 mg of EDC, stirred for 30 min, and then reacted overnight with propargylamine for the alginate-alkyne conjugate or with RGD peptide for the alginate-RGD conjugate. The mixtures were dialyzed in deionized water using a 50 kDa dialysis membrane for 4 days and freeze-dried.

### 2.3 *In vitro* characterization of imHCs encapsulated click-RGD microbeads

#### 2.3.1 Characterization of click components

The chemical compositions of the click components were analyzed by ^1^H NMR spectroscopy (BRUKER, 600 MHz, Cryo probe). PEG-azide (15 mg) was dissolved in 600 µL chloroform-D, while alginate-alkyne and alginate-RGD (15 mg each) were dissolved in 600 µL deuterium oxide. This analysis confirmed the chemical identity and purity of the reactants, ensuring the reliability of the subsequent click-RGD microbead synthesis.

#### 2.3.2 Fabrication of imHCs encapsulated click-RGD microbeads

Microbeads were fabricated using the Encapsulator B-395 Pro (BUCHI, Ireland) through an electrostatic extrusion technique (Win et al., 2024). Firstly, imHCs were cultured in DMEM/F12 medium with 10% FBS and 1% penicillin-streptomycin at 37°C in a 5% CO_2_ atmosphere. The encapsulation components listed in Supplementary Table S1 in the supporting information were dissolved in 5 mL of 0.9% sodium chloride solution and filtered with a 0.4 µm syringe filter to ensure sterility. imHCs were suspended at a concentration of 1 × 10^5^ cells/mL and extruded through a 300 µm nozzle into a 115 mM CaCl_2_ solution. The mixture was agitated for 15 min to facilitate the formation of crosslinked microbeads. The encapsulator settings were as follows: a flow rate of 7.5 mL/min, a frequency of 700 Hz, a voltage of 2.2 kV, and a stirring speed of 25%, ensuring stable microbead formation for subsequent evaluations. Microbeads were washed with sterile HEPES solution after fabrication.

#### 2.3.3 Stability assessment

An *in vitro* degradation and stability assay was performed to evaluate the structural integrity of click-RGD microbeads under physiological conditions. The 1.5% and 3% click-RGD blank microbeads without cells were suspended in a CaCl_2_ solution and incubated at 37°C with 5% CO_2_ in a humidified incubator. Their structural integrity was monitored using an inverted light microscope (Sky brand, Model: MT. 02. DS. 3). Size measurements were taken at days 0, 1, 3, 6, 10, 14, and 17 to evaluate degradation and stability. The medium was replaced every 3 days to ensure consistent conditions. For each time point, images were captured from three different fields of view, and the size of 10 microbeads per image (n = 10) was measured using ImageJ software.

#### 2.3.4 Cell viability and cell distribution of encapsulated imHCs in alginate and click-RGD microbeads

Viability and distribution of encapsulated imHCs in alginate and click-RGD microbeads were evaluated using a dual-fluorescence staining method under a confocal microscope. Two types of microbeads, imHCs encapsulated alginate microbeads and imHCs encapsulated click-RGD microbeads, were fabricated via electrostatic extrusion to encapsulate imHCs (1 × 10^5^ cells/mL). The microbeads were cultured in DMEM/F-12 medium with essential nutrients and incubated in a CO_2_ incubator. At different time points on days 0, 1, 2, 3, 4, 7, 10, and 14, the culture medium was collected, with medium replacement every 3 days. Hoechst 33,342 (6.5 μL/mL) was applied in DMEM/F12 and incubated at 37°C for 10 min. PI staining (10 μL/mL) was then performed with a 10-min incubation at room temperature in light-protected conditions. This allowed the differentiation of viable and non-viable cells. For distribution analysis, imaging was examined using a confocal microscope (LSM 800, Carl Zeiss, Jena, Germany).

#### 2.3.5 Functionality of encapsulated imHCs in alginate and click-RGD microbeads

To evaluate hepatocyte functionality, the released albumin and AFP from the encapsulated imHCs were quantified using human albumin and AFP ELISA kits. Firstly, the microbeads were cultured in a DMEM/low-glucose medium supplemented with necessary nutrients and incubated in a CO_2_ incubator. The culture supernatant was harvested on days 0, 1, 3, 7, 10, and 14 for further analysis. These assays provided a quantitative measure of hepatic function throughout the culture period.

### 2.4 Treatment of ALF in rats with imHCs encapsulated click-RGD microbeads

**FIGURE 2:**
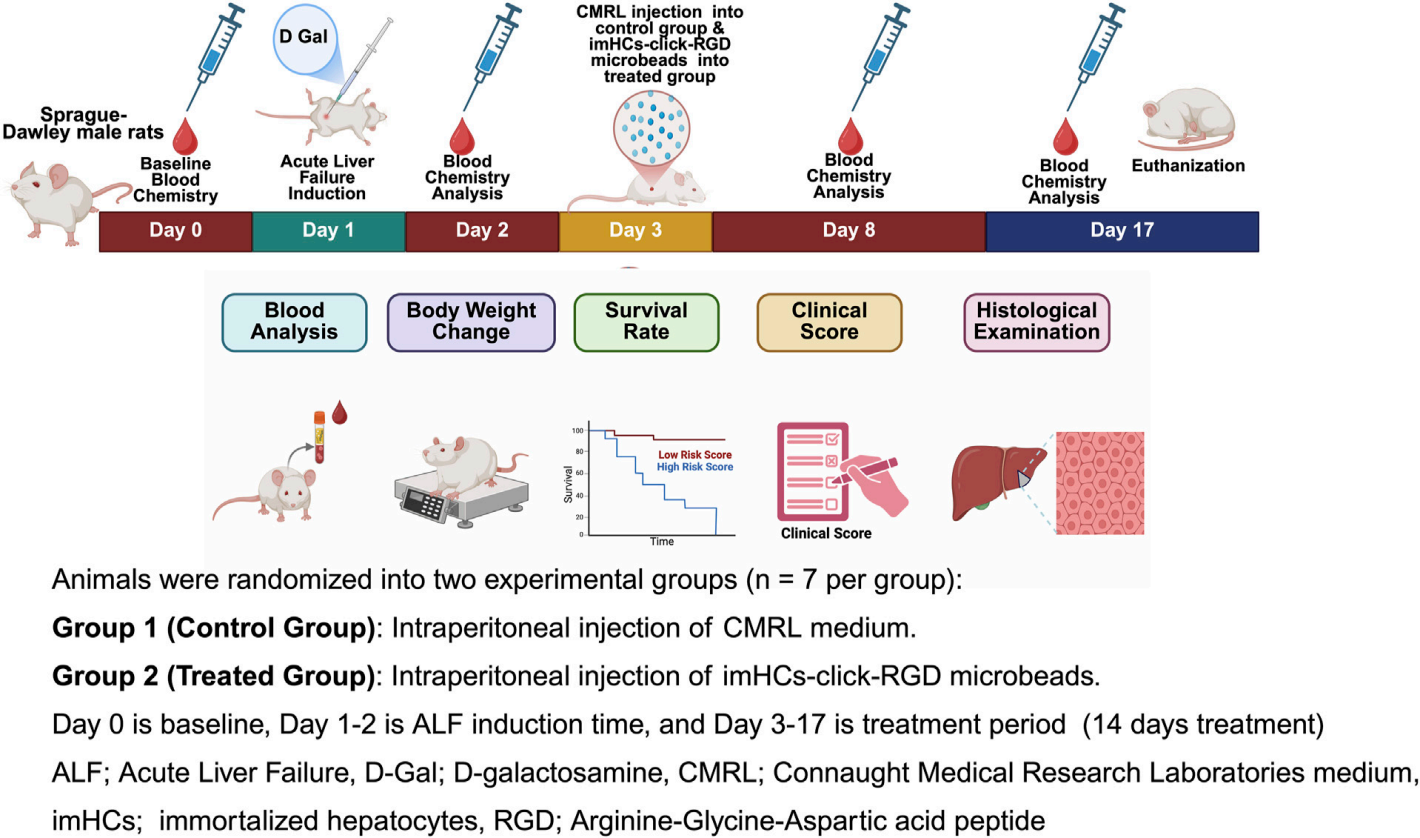
Schematic representation of the *in vivo* experimental timeline for evaluating the therapeutic efficacy of immortalized hepatocytes encapsulated in click-RGD microbeads in a D-galactosamine-induced acute liver failure rat model. Male Sprague-Dawley rats were randomly assigned to two groups (n = 7 per group): Group 1 received intraperitoneal injection of CMRL medium (control group), and Group 2 received intraperitoneal injection of imHCs-click-RGD microbeads (treated group). Day 0 represents the baseline blood chemistry assessment; Days 1–2 correspond to ALF induction via D-Gal administration; and Days 3–17 comprise the treatment period. (Created with BioRender.com).

After inducing ALF in rats with D-gal (625 mg/kg dose) according to the dose-response evaluation in the supporting information in Supplementary Figure S3, imHCs (3 × 10^6^ cells/mL) were encapsulated in click-RGD microbeads using the same method as in the *in vitro* studies before injection into rats with ALF. The microbeads were resuspended in 3 mL of CMRL medium and administered to the treated group (n = 7). The control group received 3 mL of CMRL medium. The experimental design is shown in Figure 2. On day 0, baseline blood chemistry was recorded. On day 1, D-gal was injected to induce ALF. The following day, blood chemistry analysis confirmed ALF in rats, characterized by elevated AST and ALT levels, and reduced albumin levels. On day 3, CMRL medium and imHCs encapsulated in click-RGD microbeads were injected into the animals. On days 8 and 17, blood samples (0.6 mL) were collected via a 23-gauge butterfly needle from the rats’ facial veins for blood chemistry analysis. At the study endpoint on day 17, animals were euthanized using carbon dioxide. Blood chemistry, changes in body weight, survival rate, clinical score, and histological examination were evaluated to assess the efficacy of imHCs encapsulated in click-RGD microbeads.

#### 2.4.1 Blood chemistry analysis in rats

Biochemical analysis of blood parameters was performed to monitor liver injury, assess liver function, and evaluate the therapeutic efficacy of encapsulated imHCs after transplantation. Blood samples were collected from rats on days 0, 2, 8, and 17 to assess acute liver failure, liver function, and encapsulated hepatocyte functionality after transplantation. Samples were obtained from the facial veins using a 23-gauge butterfly needle and immediately placed into heparinized and EDTA-coated tubes for biochemical analysis. After centrifugation at 3,500 rpm for 10 min, the supernatant was aliquoted and analyzed for AST, ALT, and albumin levels using the VETSCAN VS2 Chemistry Analyzer.

#### 2.4.2 Assessment of body weight change, survival rate, and clinical score in rats after treatment

To evaluate the *in vivo* therapeutic efficacy and safety of the encapsulated imHCs-click-RGD microbeads, physiological and behavioral parameters, including body weight, survival rate, and clinical score, were assessed. The survival rate provides insight into the mortality associated with the disease and treatment, offering a direct measure of the potential therapeutic benefit of the microbeads in improving survival outcomes. Weight gain indicates healthy physiological recovery in rats, reflecting the regeneration of liver function. This parameter is crucial for demonstrating the therapeutic efficacy of the imHCs encapsulated click-RGD microbeads in promoting liver regeneration and recovery from acute liver failure. The clinical score also assesses the disease severity and quantifies the treatment’s impact on the animals. This scoring system serves as a comprehensive tool to evaluate the therapeutic efficacy of the treatment and its ability to reduce clinical symptoms associated with liver failure (Boongird et al., 2011).

Body weight was recorded daily throughout the study. Toxicological effects were considered to occur if weight loss exceeded 10%, accompanied by abnormal behavior, signs of pain, or mortality. The weight recorded on day 0 was used as the baseline, and subsequent weight changes were expressed as percentage variations relative to this baseline (Mazumder et al., 2019). Weight changes were recorded on days 1 (post-D-gal injection) and 3 (post-sample injection). Additional weight monitoring occurred on days 2, 8, and 17 after blood collection for chemistry analysis. At the end of the study, rats were euthanized, and body weight changes were evaluated (Manaspon et al., 2016). In addition, the survival of the rats was monitored daily for 17 days to assess morbidity and mortality. Observations focused on treatment effects, including clinical symptoms, disease progression, and potential adverse effects. Survival data were analyzed to determine the therapeutic efficacy of imHCs-encapsulated click-RGD microbeads in enhancing hepatic recovery (Boongird et al., 2011).

Moreover, the evaluation of clinical scores is essential for quantifying the severity of liver failure and for monitoring the therapeutic impact of the administered treatment, enabling early detection of adverse effects and the overall well-being of the animals throughout the study. The clinical scores were assigned based on physiological and behavioral parameters: standard condition (score = 0), abnormal posture (1), bruising (2), distress or pain (3), significant weight loss (4), and mortality (5). This scoring system provided an objective measure of disease progression and treatment response (Boongird et al., 2011).

#### 2.4.3 Histological analysis

Histological analysis was performed to evaluate liver tissue morphology using hematoxylin and eosin staining and pathology after induction of acute liver failure and treatment (Jitraruch et al., 2014). After euthanasia, rat livers were fixed in 10% neutral buffered formalin, dehydrated through graded alcohol (70, 80, 95, and 100%), cleared with xylene, and embedded in paraffin. Tissue samples (n = 14) were oriented in molds, and paraffin blocks were sectioned (4 µm) using a rotary microtome. Sections were mounted on glass slides and air-dried. For examination, sections were deparaffinized with xylene, rehydrated, stained with hematoxylin for nuclear staining, differentiated with acid alcohol, and blued with an alkaline solution. Eosin counterstaining enhanced cytoplasmic contrast. Stained sections were dehydrated, cleared, and mounted. Microscopic evaluation was conducted using a Nikon Eclipse Ni-U light microscope to assess tissue morphology and pathological changes (Rioja et al., 2016).

#### 2.4.4 Data analysis and statistical methods

All data were expressed as mean ± standard deviation of the mean. Statistical comparisons between groups were conducted using a two-way analysis of variance (ANOVA) with repeated measures and multiple comparisons. A p-value ≤ 0.05 was considered statistically significant unless otherwise specified. The adequate sample size was calculated using G Powder software.

## 3 Results and Discussion

### 3.1 *In vitro* evaluation of imHCs encapsulated click-RGD microbeads

#### 3.1.1 Characterization of click reaction

In this study, hydroxyl-terminated PEG was functionalized with a tosyl group, which was then converted to an azide group to synthesize the click-RGD microbeads (Singh et al., 2023). After preparing PEG-azide, it was reacted with alkyne from alginate-alkyne and alginate-RGD to form a crosslinked hydrogel via CuAAC reaction (Haldón et al., 2015). Sodium ascorbate and copper (II) sulfate were added to reduce Cu^2+^ to Cu^+^, thereby facilitating the click reaction. The efficiency and functionality of this reaction were analyzed using ^1^H NMR spectroscopy, confirming the successful tosylation of PEG, conjugation of RGD to alginate, and the formation of two stable 1,2,3-triazole rings, which indicates successful crosslinking. The spectra of normal and functionalized alginate are shown in Supplementary Figures S4–S7 (Deng et al., 2021; Edward Semple et al., 2016).

#### 3.1.2 Stability assessment

**FIGURE 3:**
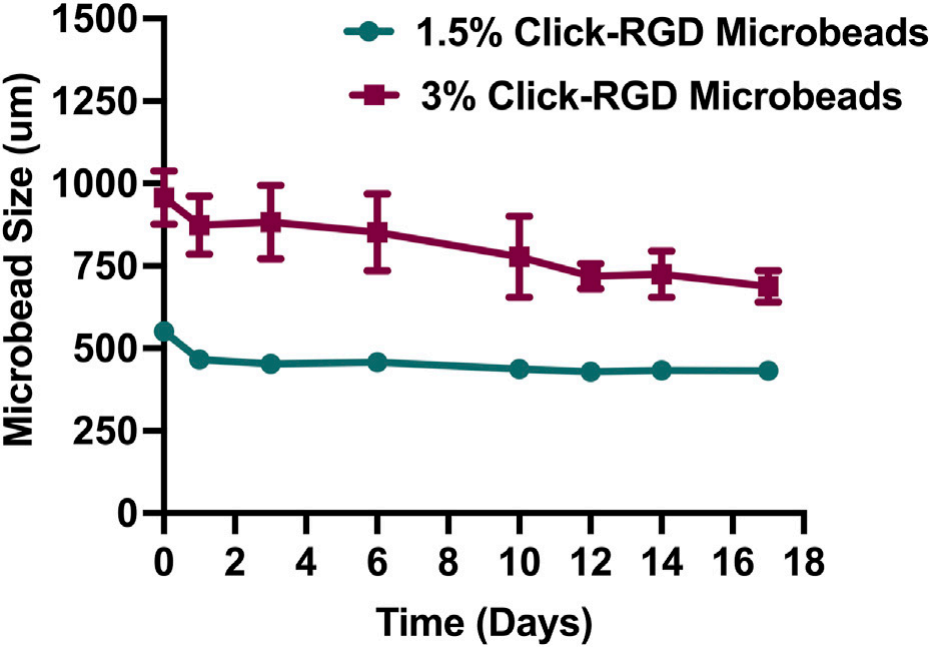
Size stability of click-RGD microbeads with 1.5% and 3% concentration without encapsulated imHCs over 14 days. Microbeads were incubated in CaCl_2_ solution, and diameters were measured at specified time points. The 3% click-RGD microbeads showed greater swelling and size fluctuation, while the 1.5% click-RGD microbeads maintained a stable diameter. Data are presented as mean ± SD (n = 10).

The stability of 1.5% and 3% click-RGD blank microbeads without cells was assessed by monitoring their size over 17 days, as shown in Figure 3. A detailed bright-field image of the microbeads is shown in Supplementary Figure S8. At day 0, the mean diameter of 1.5% microbeads was 551.12 ± 12.10 µm, while the 3% microbeads measured significantly larger at 956.63 ± 76.68 µm. By day 1, the 1.5% microbeads showed a reduction in size to 466.69 ± 11.98 µm, whereas the 3% microbeads exhibited swelling, increasing to 873.92 ± 83.62 µm. On day 3, the 1.5% group further decreased to 453.27 ± 19.41 µm, while the 3% group remained relatively stable at 883.26 ± 106.13 µm. On day 6, 1.5% microbeads expanded slightly to 457.88 ± 22.08 µm, whereas 3% microbeads decreased to 851.69 ± 110.6 µm. By day 14, both groups showed signs of stabilization, with sizes of 433.47 ± 9.42 µm and 724.77 ± 67.04 µm for 1.5% and 3% microbeads, respectively. On day 17, the final recorded sizes were 432.81 ± 12.73 µm (1.5%) and 687.80 ± 45.09 µm (3%), indicating that both formulations reached a plateau with minor fluctuations. Despite the larger and more variable size profile of the 3% microbeads, both formulations demonstrated overall stability. However, due to the potential complications associated with microbeads exceeding 500 µm for intraperitoneal injection, the 1.5% click-RGD microbeads were selected for further investigation (Dhawan et al., 2020; Gasperini et al., 2013; Mazzitelli et al., 2008; Rana et al., 2016). Statistical analysis revealed a highly significant change in microbead size over time (p < 0.0001), and all data are reported as mean ± standard deviation.

#### 3.1.3 Viability of encapsulated imHCs in click-RGD microbeads

**FIGURE 4:**
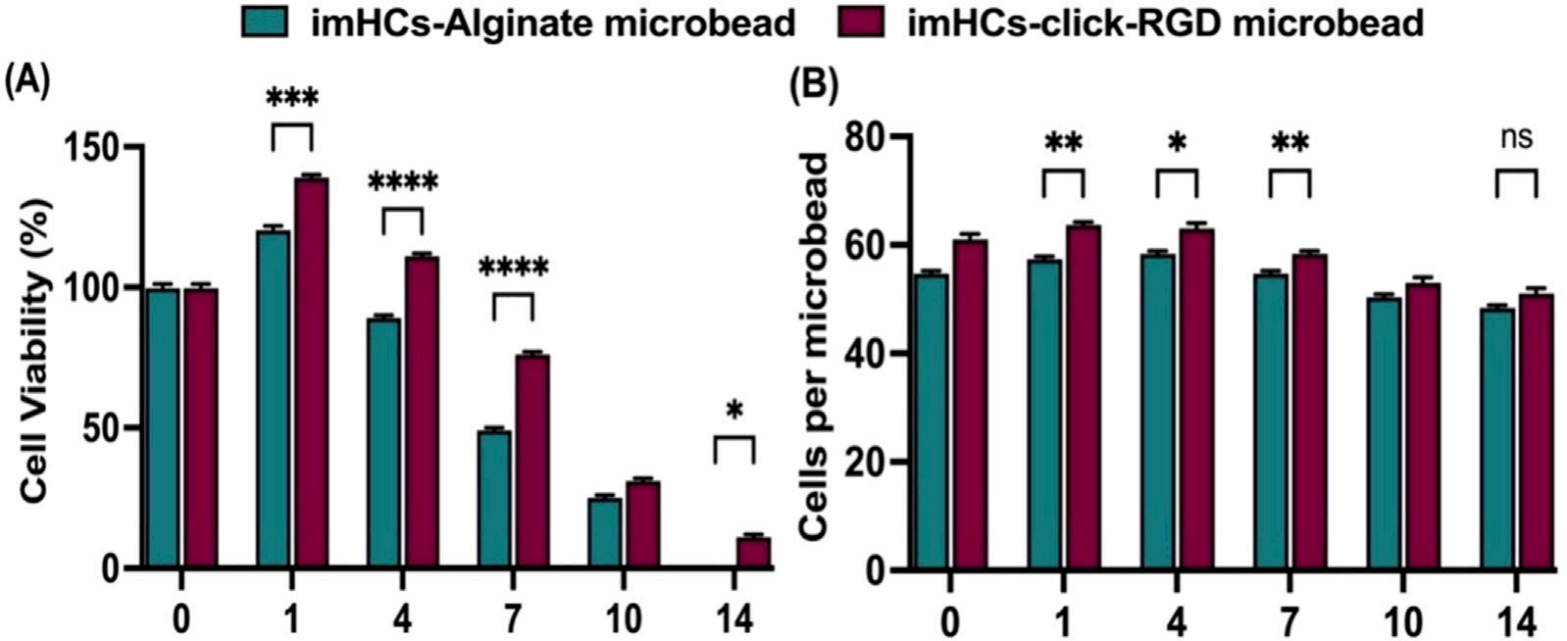
Viability and cell density of imHCs encapsulated in alginate and click-RGD microbeads. **(A)** Cell viability (%) comparison between imHCs in alginate and click-RGD microbeads at days 0–14, assessed using Hoechst and propidium iodide staining. **(B)** Comparison of cell density (cells per microbead) at corresponding time points. Data are presented as mean ± SD (n = 30). Statistical significance: *p < 0.05, **p < 0.01, ***p < 0.001, ****p < 0.0001.

The viability of imHCs encapsulated in alginate and click-RGD microbeads was evaluated by confocal microscopy using fluorescent dyes and is summarized in Figure 4A. On day 0, both groups showed nearly 100% viability. Over time, imHCs in click-RGD microbeads maintained significantly higher viability than those in alginate microbeads. In comparison, a significant difference was observed on day 1 (imHCs in click-RGD microbeads: 139% ± 0.82% vs. imHCs in alginate microbeads: 120% ± 1.25%, p < 0.001), and viability in the click-RGD group remained superior on day 4 (111% ± 0.82% vs. 89% ± 0.82%; p < 0.0001), on day 7 (76% ± 0.82% vs. 49% ± 0.82%; p < 0.0001), and on day 14 (11% ± 0.82% vs. 0%; p < 0.05). The enhanced viability is attributed to improved cell adhesion in click-RGD microbeads. Figure 4B presents the number of cells per microbead. The click-RGD microbeads exhibited consistently higher cell counts than the alginate microbeads at all the time points, with statistically significant differences observed on days 1, 4, and 7 (p < 0.01 and p < 0.05). Although a substantial difference persisted on day 14 (p = 0.05), the magnitude of the difference was minimal. Confocal images showed more uniform cell distribution in click-RGD microbeads in Supplementary Figure S9. These findings indicate that click-RGD microbeads enhance imHCs’ viability and retention, supporting their potential for liver cell therapy and regenerative medicine.

#### 3.1.4 Functionality of encapsulated imHCs in the microbeads

To assess the hepatic functionality of encapsulated imHCs, the secretion levels of albumin and AFP were quantified, as these proteins serve as critical indicators of liver-specific metabolic activity and cellular maturation. Albumin, a primary plasma protein synthesized by hepatocytes, plays a crucial role in maintaining colloidal osmotic pressure and facilitating the transport of both endogenous and exogenous compounds. AFP, a well-established marker of fetal liver development, reflects the differentiation status and early hepatic lineage commitment of hepatocyte-like cells (Jeng et al., 2023; Sala-Trepat et al., 1979).

**FIGURE 5:**
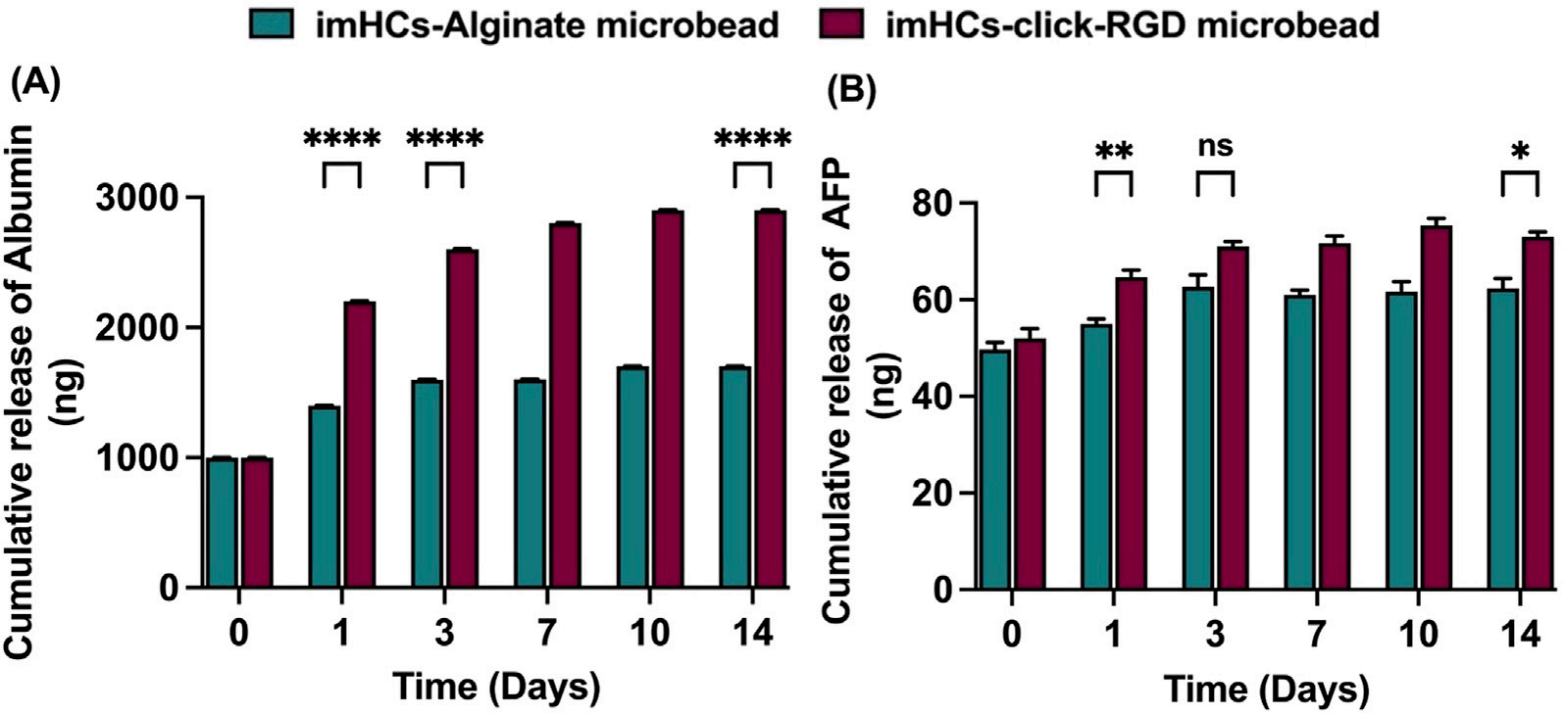
Cumulative release of hepatic markers from imHCs encapsulated in alginate and click-RGD microbeads. **(A)** Albumin release was significantly higher in the click-RGD microbeads group on days 1, 3, and 14, indicating enhanced hepatic function. **(B)** AFP release was also elevated in the click-RGD group, with significant differences on days 1 and 14. Data are presented as mean ± SD (n = 3). Statistical significance: *p < 0.05, **p < 0.01, ***p < 0.001, ****p < 0.0001.

The cumulative release of albumin and AFP from imHCs encapsulated in alginate and click-RGD microbeads was assessed on days 1, 3, 7, 10, and 14. Albumin secretion was significantly elevated in the click-RGD microbeads group compared to the alginate microbeads group at all assessed time points, indicating enhanced hepatic functionality in Figure 5A. On day 1, albumin levels were 1,399 ± 0.82 ng from the alginate microbeads compared to 2,202 ± 1.63 ng from the click-RGD microbeads (p < 0.0001). This trend continued on day 3 (1,599.67 ± 1.25 vs. 2,602.33 ± 2.05 ng, p < 0.0001) and day 14 (1702.33 ± 1.7 vs. 2,901 ± 2.16 ng, p < 0.0001).

AFP secretion was consistently higher in the click-RGD microbeads group than in the alginate group in Figure 5B. On day 1, AFP levels were significantly elevated in the click-RGD group (64.67 ± 1.25 ng) relative to alginate (55 ± 0.82 ng; p < 0.01). By day 3, the difference between the groups was not statistically significant (71 ± 0.82 ng vs. 62.67 ± 2.05 ng; p = 0.3). However, on day 14, AFP secretion was again significantly higher in the click-RGD group (73 ± 0.82 ng) compared to alginate (62.33 ± 1.7 ng; p < 0.05). These findings demonstrate that encapsulation of imHCs in click-RGD microbeads significantly enhances albumin and AFP secretion compared to alginate microbeads, likely due to improved cell-matrix interactions from PEG and RGD modification. The click-RGD system provides a more supportive microenvironment for imHCs, showing strong potential for liver cell-based therapies.

### 3.2 Treatment of ALF in rats with imHCs encapsulated click-RGD microbeads

In this study, seven male Sprague-Dawley rats, aged 8–12 weeks and weighing between 300–480 g, were used in each experimental group. Rats were acclimated for 3–5 days before the experiment and maintained under standardized conditions: a 12-h light/dark cycle, a temperature of 22°C ± 3°C, and humidity between 30% and 70%. They had continuous access to food and 5–7 ppm chlorinated water. Body weight was monitored daily throughout the study. The research adhered to the guidelines of the Mahidol University National Laboratory Animal Centre (Ethical approval: MUSC66-048-678) and the National Institutes of Health’s protocol for the care and use of laboratory animals. A D-galactosamine (D-gal) dose-response study was conducted to establish an effective concentration for inducing ALF in rats, as illustrated in Supplementary Figure S3.

#### 3.2.1 Evaluation of hepatic biomarkers after injection with CMRL medium and imHCs encapsulated click-RGD microbeads

**FIGURE 6:**
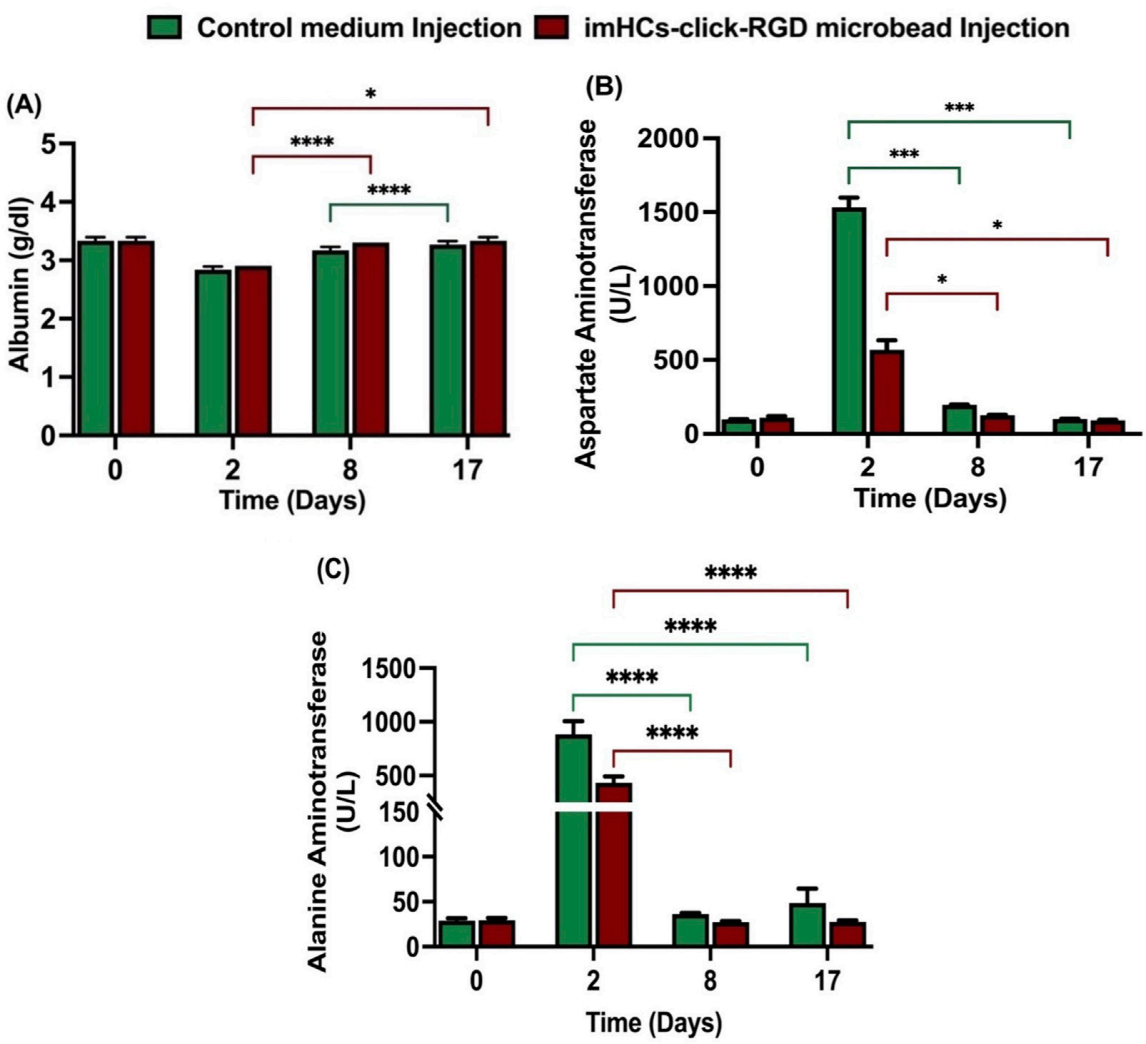
Serum biochemical analysis of liver function following CMRL medium or imHCs-click microbeads injection in D-gal-induced rats. **(A)** Serum albumin levels were stable in both groups but increased significantly in the treated group by day 8. **(B)** AST levels peaked on day 2 in the CMRL group, indicating acute liver injury, and decreased significantly in the treated group. **(C)** ALT levels increased on day 2 in the control group, while the treated group showed significantly lower ALT levels on days 2, 8, and 17, indicating improved recovery. Data are presented as mean ± SD (n = 3). Statistical significance: *p < 0.05, **p < 0.01, ***p < 0.001, ****p < 0.0001.

After the injection of medium and microbeads (400-700 μm) in Supplementary Figure S10, blood chemistry was assessed in both the CMRL medium-injected rats (control group) and imHCs-encapsulated click-RGD microbead injected rats (treated group) at days 0, 2, 8, and 17, with albumin, AST, and ALT levels serving as markers for liver function in Figure 6. The reference ranges for albumin (3.2–4.62 g/dL), AST (94.34–228.28 U/L), and ALT (9.78–50.55 U/L) are used as a baseline for evaluating hepatocellular integrity and functionality (Mazumder et al., 2019). On day 0, baseline albumin levels (control group: 3.3 ± 0.05 g/dL, treated group: 3.3 ± 0.05 g/dL) showed no significant differences. By day 2, both groups exhibited a decrease in albumin levels (control group: 2.83 ± 0.05 g/dL, treated group: 2.9 ± 0.05 g/dL), reflecting acute liver injury. On day 8, the treated group showed significantly higher albumin levels (3.3 ± 0.1 g/dL, p < 0.0001) compared to the control group (3.10 ± 0.1 g/dL, not significant), indicating an improved hepatic function in the treated group after implantation of microbeads. By day 17, the treated group maintained stable albumin levels (3.33 ± 0.05 g/dL, p < 0.05), while the control group showed a slightly increased albumin level (3.20 ± 0.05 g/dL, p < 0.0001).

AST levels were nearly similar between the groups on day 0 (control group: 97 ± 1.14 U/L, treated group: 101 ± 12.3 U/L). On day 2, AST increased significantly in the control group (1,532 ± 54.68 U/L) compared to the treated group (568.33 ± 53.39 U/L), indicating the presence of acute liver injury. By day 8, AST levels decreased in both groups (control group: 195.92 ± 1.3 U/L, p < 0.001; treated group: 125.33 ± 2.49 U/L, p < 0.05), with the treated group showing improved liver function. On day 17, both groups showed decreased AST levels (control group: 101.67 ± 0.94 U/L, p < 0.001; treated group: 91.33 ± 2.49 U/L, p < 0.05), with the treated group showing better recovery.

ALT levels were similar on day 0 (control group: 29 ± 2.2 U/L, treated group: 29.3 ± 2.1 U/L). After the D-gal injection on day 2, the control group exhibited a significant rise in ALT (883.3 ± 99.9 U/L) compared to the treated group (433.7 ± 47.6 U/L), indicating liver damage. On day 8, ALT levels in the treated group (27.3 ± 0.9 U/L) were lower than the control group (36.3 ± 0.9 U/L), indicating better recovery. By day 17, ALT levels in the control group significantly increased to (48.7 ± 13 U/L), whereas the treated group exhibited a consistent reduction to (27.7 ± 1.2 U/L). All differences were statistically significant (p < 0.05), demonstrating the therapeutic efficacy of encapsulated microbeads in promoting liver recovery.

The treated group consistently showed better recovery of hepatic biomarkers, including significantly lower AST and ALT levels and sustained albumin production, compared to the control group. These results demonstrate that imHCs-encapsulated click-RGD microbeads effectively attenuate liver injury and support hepatic regeneration in the D-galactosamine-induced acute liver failure model.

#### 3.2.2 Survival rate and body weight change (%) and clinical score

**FIGURE 7:**
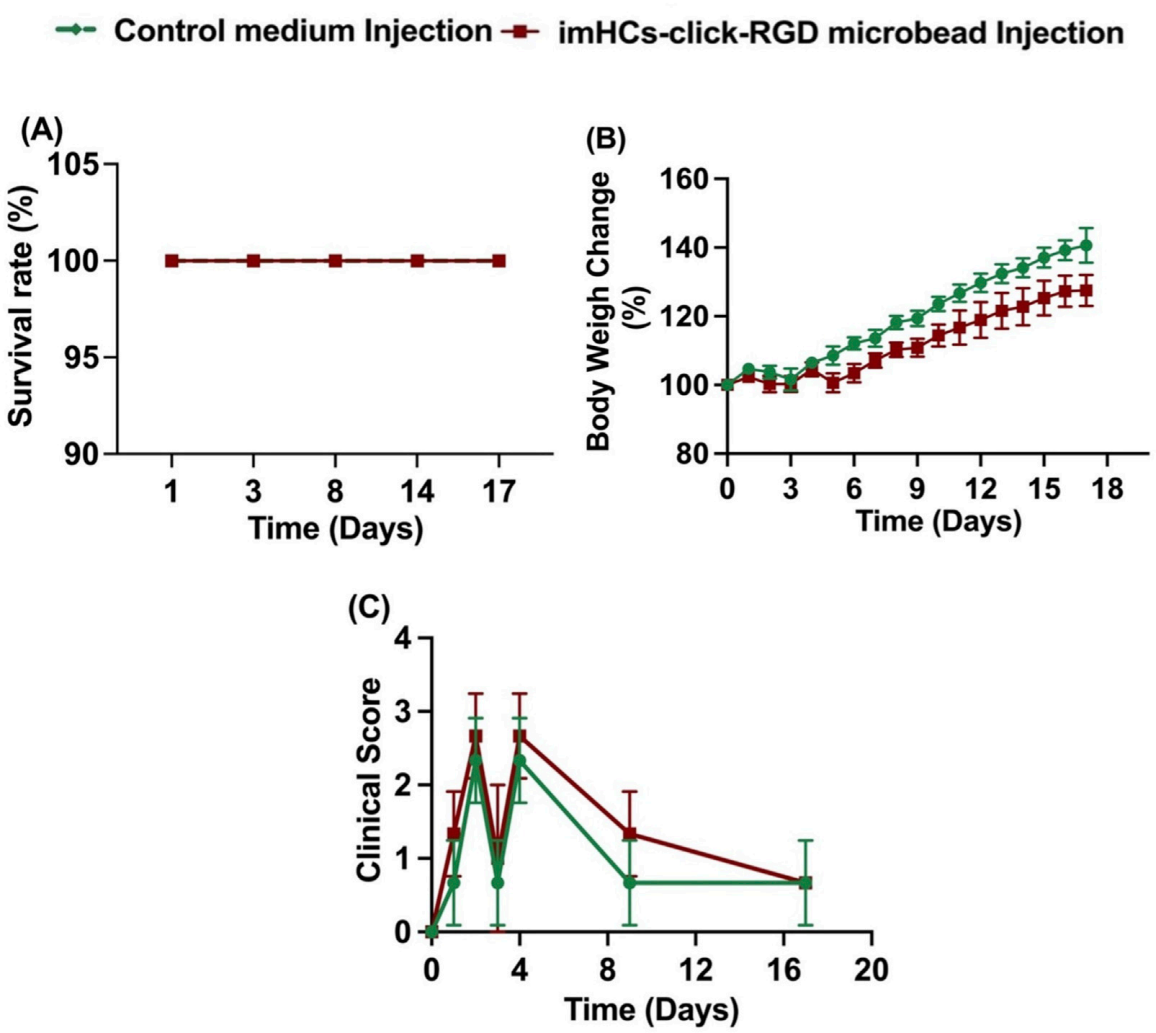
Survival rate, body weight change, and clinical score were evaluated in rats treated with CMRL medium injection (control group) and imHCs-click microbeads injection (treated group). **(A)** Survival rate, **(B)** Body weight change, and **(C)** Clinical score over time (n = 3). Data are presented as mean ± standard deviation.

This study assessed survival rate, body weight change, and clinical score on days 0, 2, 8, and 17 after the injection of CMRL medium in the control group and imHCs-encapsulated click-RGD microbeads in the treated group in Figure 7. Clinical scores, ranging from 0 (normal) to 5 (death), were used to assess disease severity and treatment response. The scoring system was based on predefined physiological and behavioral parameters as follows: standard condition (score = 0), abnormal posture (score = 1), presence of bruising (score = 2), signs of distress and pain (score = 3), significant weight loss (score = 4), and mortality (score = 5). (Boongird et al., 2011).

At day 0, before injection, both groups began with comparable baseline parameters across all measured indices, including survival rate (100%), body weight (100%), and clinical score (0), indicating similar initial physiological states. By day 2, after the D-gal injection, both groups maintained complete survival. However, differences in clinical scores emerged: the treated group exhibited a slightly higher clinical score than the control group, suggesting increased physiological stress or symptom severity. Body weight changes were minimal and similar between groups at this early time. On day 8, after the treatment, the survival rate remained 100% for both groups. The control group showed a more significant increase in body weight (120%) compared to the treated group (112%, p < 0.01), suggesting better overall recovery and growth. Clinical scores decreased in both groups; however, the control group demonstrated a more rapid reduction in symptoms, with scores closer to baseline than those of the treated group. By day 17, survival continued to be unaffected in both groups. The divergence in body weight gain became more pronounced: the control group approached a 140% increase, whereas the treated group reached only 125% (p < 0.0001). Clinical scores further declined in both groups, with the control group maintaining lower scores, indicating sustained recovery, while the treated group still presented with mild residual symptoms in some animals.

Both groups maintained full survival throughout the study period, indicating the safety of the treatment. Although the treated group exhibited slightly delayed weight gain and higher clinical scores during the early recovery phase, these parameters gradually improved over time. The persistent but mild symptoms in the treated group may reflect ongoing hepatic regeneration facilitated by the transplanted encapsulated imHCs. These findings suggest that imHCs-encapsulated click-RGD microbeads are a safe and potentially effective therapeutic approach, supporting gradual recovery from acute liver failure.

#### 3.2.3 Histological evaluation of liver tissue

**FIGURE 8:**
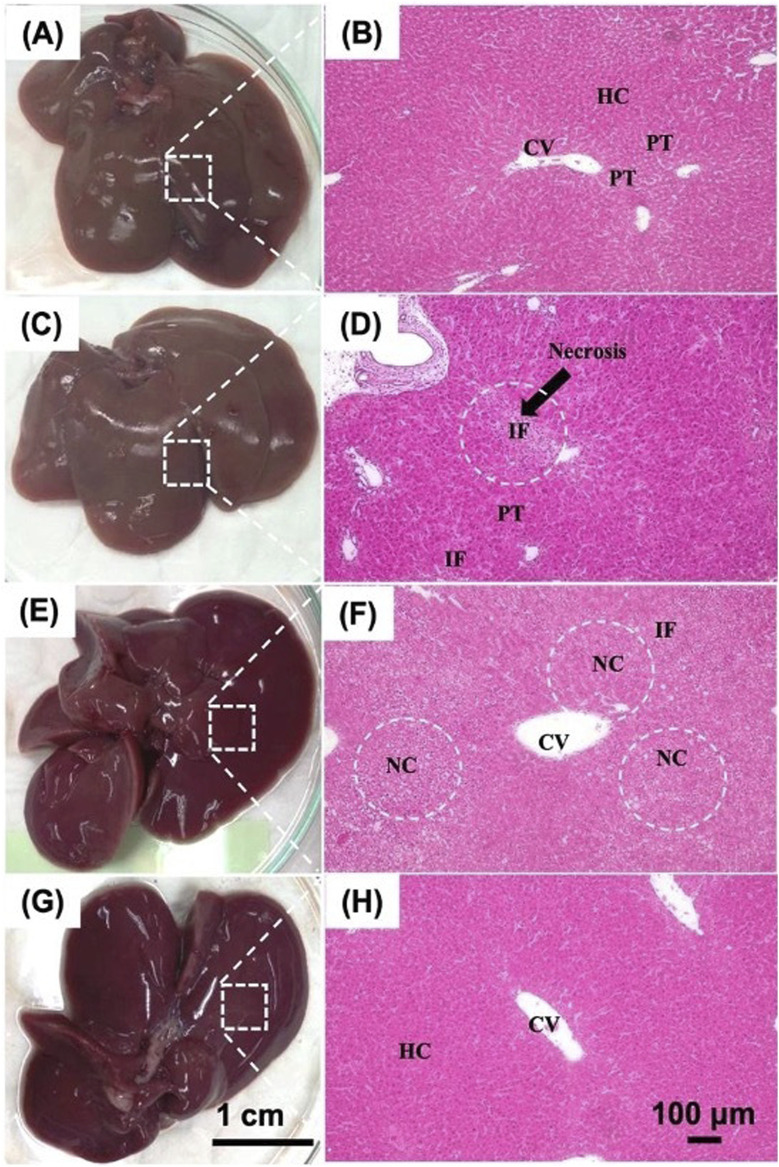
Histological evaluation of liver tissues in D-gal-induced ALF rats after intraperitoneal transplantation of imHCs encapsulated in click-RGD microbeads. **(A,B)** Normal liver morphology with intact architecture, hepatocytes (HC), central vein (CV), and portal triads (PT). **(C,D)** ALF rats show inflammatory cell infiltration (IF) and portal triads (PT). **(E,F)** Rats administered CMRL medium post-D-gal show moderate necrosis and inflammation, characterized by hepatocellular necrosis (NC) and inflammatory cell infiltration (IF). **(G,H)** Rats treated with imHCs-click-RGD microbeads demonstrate enhanced liver architecture, reduced necrosis and inflammation, and preserved normal hepatocytes (HC) and the central vein (CV). Images taken with H&E staining at 100× magnification. Scale bars: 1 cm (gross morphology), 100 µm (histology).

Histological analysis of liver tissue using H&E staining was performed at the study endpoint, as shown in Figure 8. Four groups were compared: (1) negative control rats with normal liver morphology, (2) positive control rats with D-gal injection, (3) control group rats receiving CMRL medium, and (4) treated rats with imHCs-encapsulated click-RGD microbeads. The negative control group exhibited well-preserved liver architecture, with intact hepatocytes, sinusoids, and portal triads, indicating normal function. In contrast, the positive control group showed severe liver disruption, including necrosis, inflammatory cell infiltration, and apoptotic cells, confirming acute liver failure (Chen and Xu, 2014; Zhang et al., 2011). The CMRL medium-injected group displayed partial recovery but showed persistent necrosis and structural abnormalities, indicating incomplete regeneration (Éboli et al., 2016). The treated group demonstrated substantial liver regeneration, characterized by restored architecture, well-organized lobules, and reduced inflammation. These histological findings suggest that imHCs-encapsulated click-RGD microbeads effectively promote liver regeneration after acute liver failure, offering potential as a therapeutic strategy for liver tissue repair.

## 4 Discussion

Compared to conventional therapeutic strategies for acute liver failure, such as orthotopic liver transplantation, our approach offers a less invasive and potentially more scalable solution. While liver transplantation remains the gold standard, it is limited by donor shortages, high costs, and the need for lifelong immunosuppression (Dhawan et al., 2020; Jitraruch et al., 2014). Although the encapsulation of primary hepatocytes in alginate microbeads has shown therapeutic promise in the treatment of acute liver failure, their clinical applicability is often hindered by poor post-transplant cell viability, inadequate mechanical stability of the encapsulation matrix, and a rapid decline in hepatocyte-specific functions *in vivo* (Yu et al., 2012). In contrast, our click-RGD microbeads demonstrated enhanced biocompatibility, improved cell viability, and significantly higher albumin and AFP secretion *in vitro,* as well as supporting liver function *in vivo*. Notably, the imHCs used in this study offer a consistent and renewable cell source compared to primary hepatocytes, which are limited and highly variable. Throughout this period, no signs of abnormal tissue growth, tumor formation, or cellular overproliferation were observed in the peritoneal cavity or on the liver surface by gross anatomical and histological assessment. Nonetheless, the rigorous evaluation of genetic stability, karyotype integrity, and tumorigenic potential is essential before considering clinical translation. Moreover, the inclusion of RGD peptides facilitated better cell-matrix interactions compared to alginate microbeads in Supplementary Figure S12, while the click-chemistry crosslinking ensured structural stability and minimal immunogenic response (Magalhães et al., 2024).

In this study, intraperitoneal (IP) injection was intentionally selected as a localized delivery method to place the encapsulated imHCs click-RGD microbeads in proximity to the liver, allowing them to support hepatic function during the regeneration phase after acute liver failure. The aim was to assess local therapeutic efficacy and biocompatibility rather than systemic biodistribution. While clinical translation may require intraportal or intrahepatic administration, IP delivery in rodent models provides a reproducible and practical route for initial *in vivo* evaluation of encapsulated constructs. Notably, recent reports (Dhawan et al., 2020; Fitzpatrick et al., 2023) demonstrate that IP delivery of hepatocyte-loaded microbeads has progressed into early-phase clinical trials, showing safety, tolerability, and preliminary efficacy in patients with acute liver failure.

In microbead fabrication, the click-functionalized components (PEG-azide, alginate-alkyne, and alginate-RGD) are extruded into a calcium chloride bath; however, the primary crosslinking mechanism is covalent, initiated by Cu(I)-catalyzed azide-alkyne cycloaddition (CuAAC). Although transient ionic interactions may occur during bead formation, we minimized residual calcium by thoroughly washing the microbeads twice with autoclaved deionized water. Importantly, no calcification or signs of inflammation were observed at the implantation site by gross anatomy or histological analysis. The microbeads also fully degraded within 14 days, indicating minimal long-term calcium retention or toxicity.

For biocompatible materials, the acute inflammatory phase is followed by a brief chronic inflammatory response, primarily involving mononuclear cells, which typically resolves within approximately 2 weeks (Anderson et al., 2008). This study was conducted as a short-term *in vivo* evaluation to investigate the biocompatibility and therapeutic efficacy of imHCs-encapsulated click-RGD microbeads in a rat model of acute liver failure. The microbeads were specifically engineered to provide temporary, localized hepatic support during the liver regeneration process and to undergo complete biodegradation within a 14-day implantation period, as shown in Supplementary Figure S11. In line with this design, neither fibrotic tissue formation nor residual microbead material was observed on the liver surface during gross anatomical examination, and no evidence of fibrosis was detected in histological analysis at the study endpoint. These findings indicate favorable short-term biocompatibility and functional integration. Nevertheless, it is recognized that the current timeframe may be insufficient to capture delayed immunological reactions, fibrotic encapsulation, and prolonged tissue remodeling. As such, future studies will include extended implantation periods to thoroughly evaluate chronic host responses, the kinetics of microbead degradation, and the progression of tissue remodeling.

Future work will incorporate bioluminescence imaging using luciferase-expressing encapsulated imHCs and *in vivo* Imaging System (IVIS) to non-invasively track cell localization and viability. (Strohschein et al., 2015). This will clarify whether transplanted cells migrate to the liver or act locally in the peritoneal cavity. Additionally, vinculin immunostaining will be used to assess focal adhesion and integrin-RGD interactions, offering insight into cell-matrix engagement and the role of RGD in enhancing cell retention (Re’em et al., 2010). Future studies should integrate *in vivo* tumorigenicity assays, extended implantation durations, and comprehensive genomic analyses to further assess the long-term safety profile of imHCs.

## 5 Conclusion

This study demonstrated the therapeutic potential of imHCs-encapsulated click-RGD microbeads in a D-galactosamine-induced acute liver failure rat model. Compared to conventional alginate microbeads, the click-RGD system showed enhanced *in vitro* cell viability, uniform distribution, and sustained albumin and AFP secretion. *In vivo*, the microbeads improved liver function (lower AST and ALT), supported hepatic regeneration, and fully degraded without adverse effects. Histological analysis confirmed restored liver architecture and reduced inflammation, whereas controls showed fibrosis and immune infiltration. These findings support the use of click-RGD microbeads as a biocompatible and functional platform for bridging liver regeneration or transplantation. Future work should investigate long-term efficacy, immune response, and co-encapsulation with stem cells and growth factors to further enhance therapeutic outcomes.

## Data Availability

The original contributions presented in the study are included in the article/Supplementary Material, further inquiries can be directed to the corresponding author.
