# Supplementary material for: In vitro and in vivo evaluation of immortalized hepatocyte encapsulated click-microbeads with RGD peptide for treatment of liver failure in male rats
Source: Front Bioeng Biotechnol. 2025 Jul 15;13:1629228. doi: 10.3389/fbioe.2025.1629228 (PMC12303912; doi:10.3389/fbioe.2025.1629228)
Supplement: Supplementary file 1 [file DataSheet1.docx]

**Supplementary Materials**

***In vitro* and *in vivo* evaluation of Immortalized Hepatocyte Encapsulated Click-microbeads with RGD peptide for Treatment of Liver Failure in male rats**

Su Yee Win^1,†^, Pinunta Nittayacharn^1,†^ , Jatupoom Ngernmark^1^, Mongkol Chavalitsarot^1^, Chitinart Thedrattanawong^1^, Khanit Sa-ntgiamsuntorn^2^, Suradej Hongeng^3^ and Norased Nasongkla^1*^

^1^Department of Biomedical Engineering, Faculty of Engineering, Mahidol University, Nakhon Pathom, 73170, Thailand.

^2^Department of Biochemistry, Faculty of Pharmacy, Mahidol University, Bangkok, 10400, Thailand.

^3^Department of Pediatrics, Faculty of Medicine, Ramathibodi Hospital, Mahidol University, Bangkok, 10400, Thailand.

*Corresponding author: Prof. Norased Nasongkla, PhD

Department of Biomedical Engineering, Faculty of Engineering, Mahidol University, Nakhon Pathom, 73170, Thailand

norased.nas@mahidol.ac.th

^†^These authors contributed equally to this manuscript.

**Table 1.** **Initial substances for the preparation of microbeads**

| **Formulations** | **Initial substances for 5 mL solution (mg)** | | | |
| --- | --- | --- | --- | --- |
|  | **Alginate** | **Alginate-alkyne** | **Azide-PEG-Azide** | **Alginate-RGD** |
| Alginate microbeads | 75 | - | - | - |
| Click-RGD microbeads | - | 14.05 | 425.15 | 60.95 |

**1. Click reaction.**


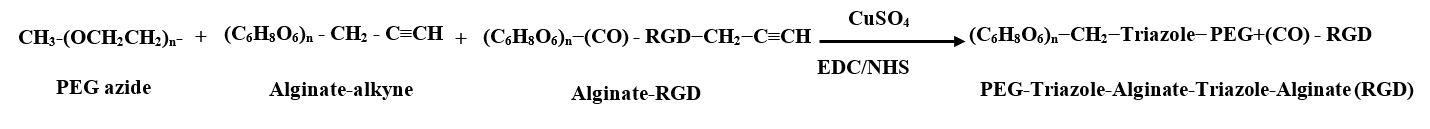
 PEG-azide was synthesized to introduce azide (-N₃) groups, enabling the copper-catalyzed azide-alkyne cycloaddition (CuAAC) reaction. Alginate derivatives, including alginate-alkyne and alginate-RGD, were prepared to incorporate alkyne (-C≡CH) groups and RGD peptides, respectively. These modifications facilitated biofunctionalization and crosslinking through CuAAC. The conjugation of PEG-azide with alginate-alkyne and alginate-RGD resulted in the formation of two triazole rings (z), confirming the successful execution of the click reaction. (Edward Semple et al., 2016)


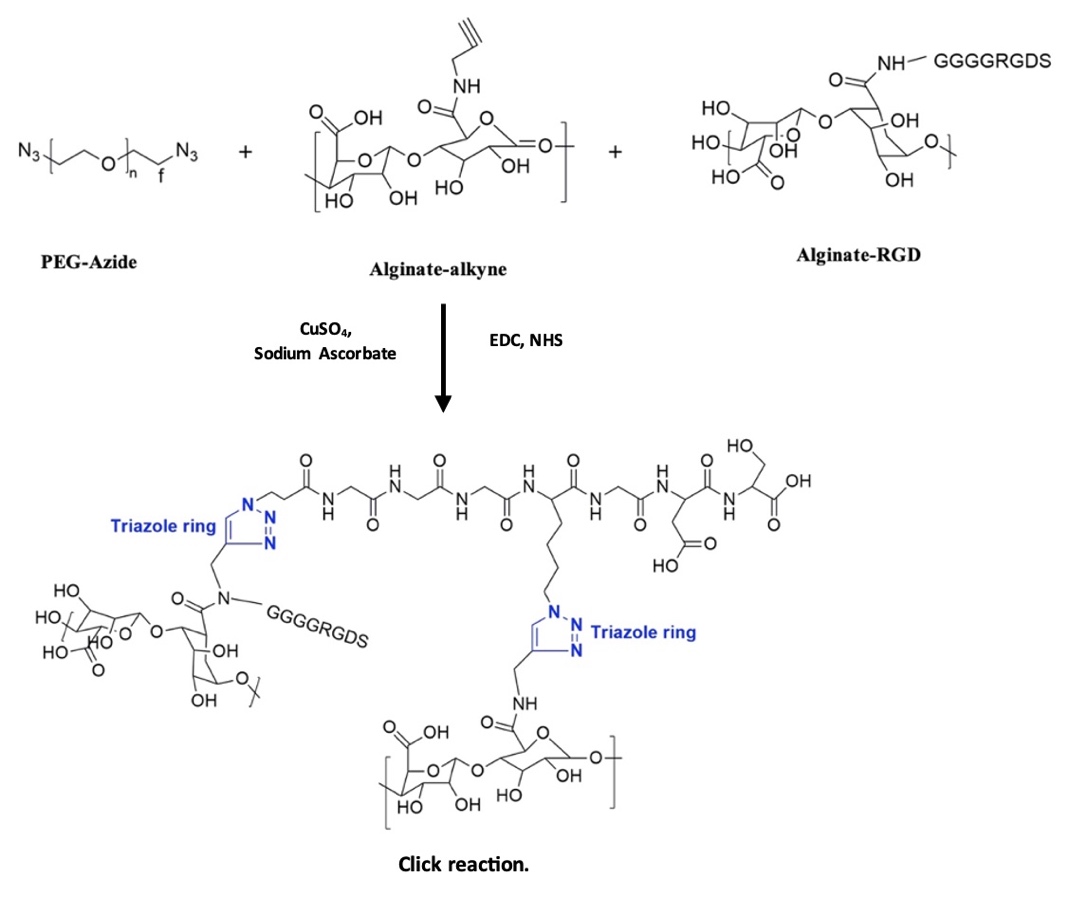


**Figure S1.** Schematic illustration of the conjugation process forming a click-crosslinked alginate hydrogel network. PEG-azide and alginate-alkyne are conjugated via copper(I)-catalyzed azide-alkyne cycloaddition using CuSO₄ and sodium ascorbate, resulting in the formation of stable 1,2,3-triazole linkages within the hydrogel matrix. Simultaneously, alginate is functionalized with the RGD peptide via EDC/NHS-mediated coupling to introduce RGD motifs for cell adhesion. The resulting biofunctional hydrogel combines structural integrity with integrin-binding bioactivity.

**
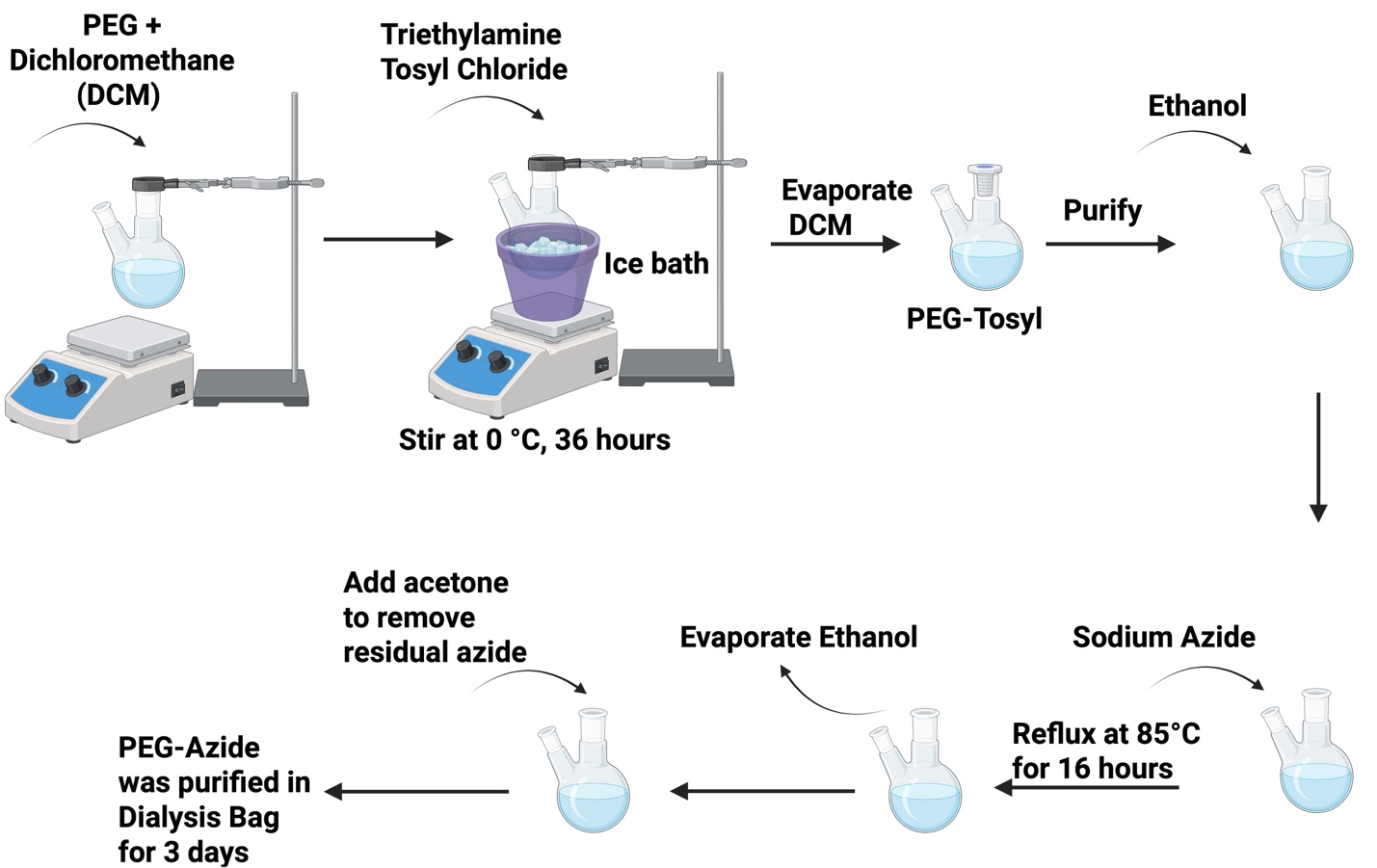
Figure S2.** Synthesis of PEG-azide via tosylation and azidation. PEG was first functionalized with a tosyl group, then converted to PEG-azide through nucleophilic substitution with sodium azide. This two-step process enables the introduction of azide functionality for subsequent click chemistry applications.

**2. Optimization of D-galactosamine Dose for Induction of Acute Liver Failure (ALF) in Rats**

The dose-response assessment of D-gal was conducted to determine an effective dose for inducing ALF in rats, thereby facilitating subsequent investigations into liver dysfunction and potential therapeutic interventions. D-gal is frequently used to produce ALF in rats due to its hepatotoxic effects. The mechanism of action of D-gal is the disruption of hepatic cell metabolism, resulting in cellular apoptosis and hepatic dysfunction. In this study, doses of D-gal ranging from 250 to 1000 mg/kg were administered to determine the optimal dose for inducing ALF in the test animals. After the administration of varying doses of D-gal, the blood chemistry of the rats, including levels of albumin, AST, and ALT, was assessed using a blood autoanalyzer (VETSCAN VS2 Chemistry Analyzer) to confirm the induction of ALF in rats.

The reference ranges for albumin (3.2-4.62 g/dL), aspartate aminotransferase (AST; 94.34-228.28 U/L), and alanine aminotransferase (ALT; 9.78-50.55 U/L) serve as benchmarks for evaluating hepatocellular integrity and function. (Patel et al., 2024) In this study, doses of 250 and 500 mg/kg did not induce ALF, as evidenced by normal AST, ALT, and albumin levels. In contrast, D-galactosamine doses of 750 and 1000 mg/kg successfully induced acute liver failure, as evidenced by marked elevations in serum AST and ALT levels and a concomitant reduction in albumin concentration. However, both doses were associated with high toxicity, leading to mortality within 72 hours post-administration. (Chen et al., 2024; Rouf et al., 2021; Yang et al., 2021) 625 mg/kg was tested to identify a sublethal dose. This dose caused significant liver injury, elevated AST and ALT, and reduced albumin without immediate lethality. Rats experienced 5% transient weight loss and survived up to 17 days, confirming 625 mg/kg as a reliable dose for ALF induction in this study.

**~~
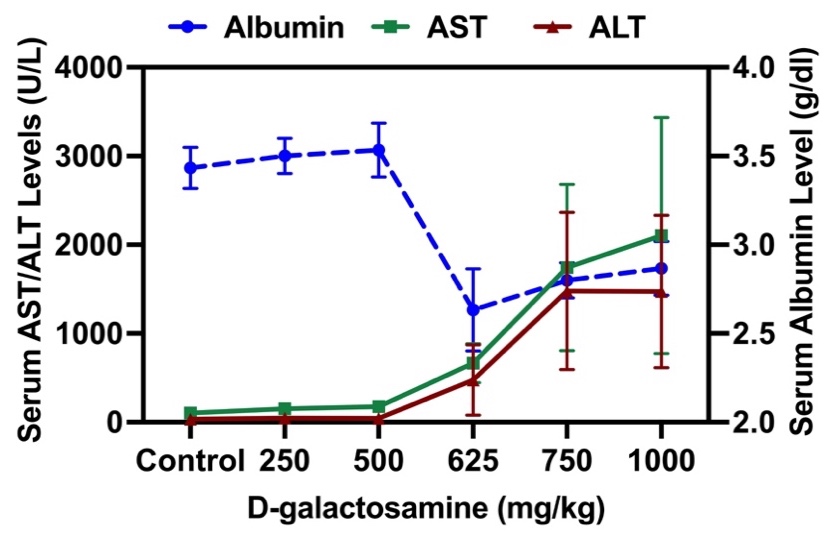
~~**

**Figure S3.** Evaluation of serum albumin, AST, and ALT levels after different doses of D-galactosamine injection (250-1000 mg/kg) to induce acute liver failure in rats.

**
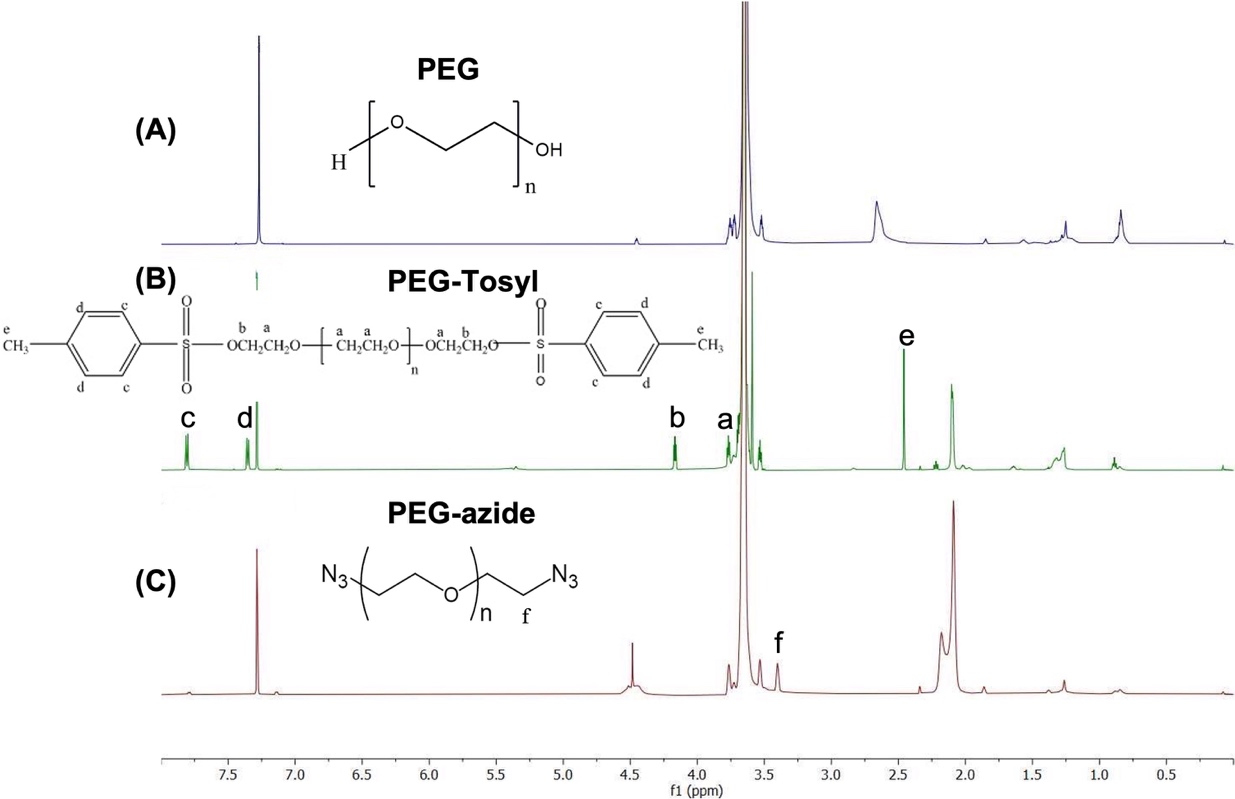
**

**Figure S4. ¹**H NMR spectra confirm the stepwise synthesis of PEG-tosyl and PEG-azide, demonstrating successful functionalization. (A) Unmodified PEG peaks at 3.6 ppm (a), representing the PEG backbone. (B) PEG-tosyl spectrum displays peaks at 4.14 ppm (b) for the crosslinker and aromatic proton signals at 7.78 ppm (c), 7.3 ppm (d), and 2.43 ppm (e), indicating successful tosylation, with a functionalization efficiency of 74.22%. (C) The PEG-azide spectrum shows the absence of tosyl-related peaks (c, d) and the appearance of a peak at 3.33 ppm (f), confirming the azide substitution. The functionalization efficiency for azide was 72.71%. These results validate the effective synthesis and structural modification of PEG derivatives, ensuring their readiness for further functionalization processes.


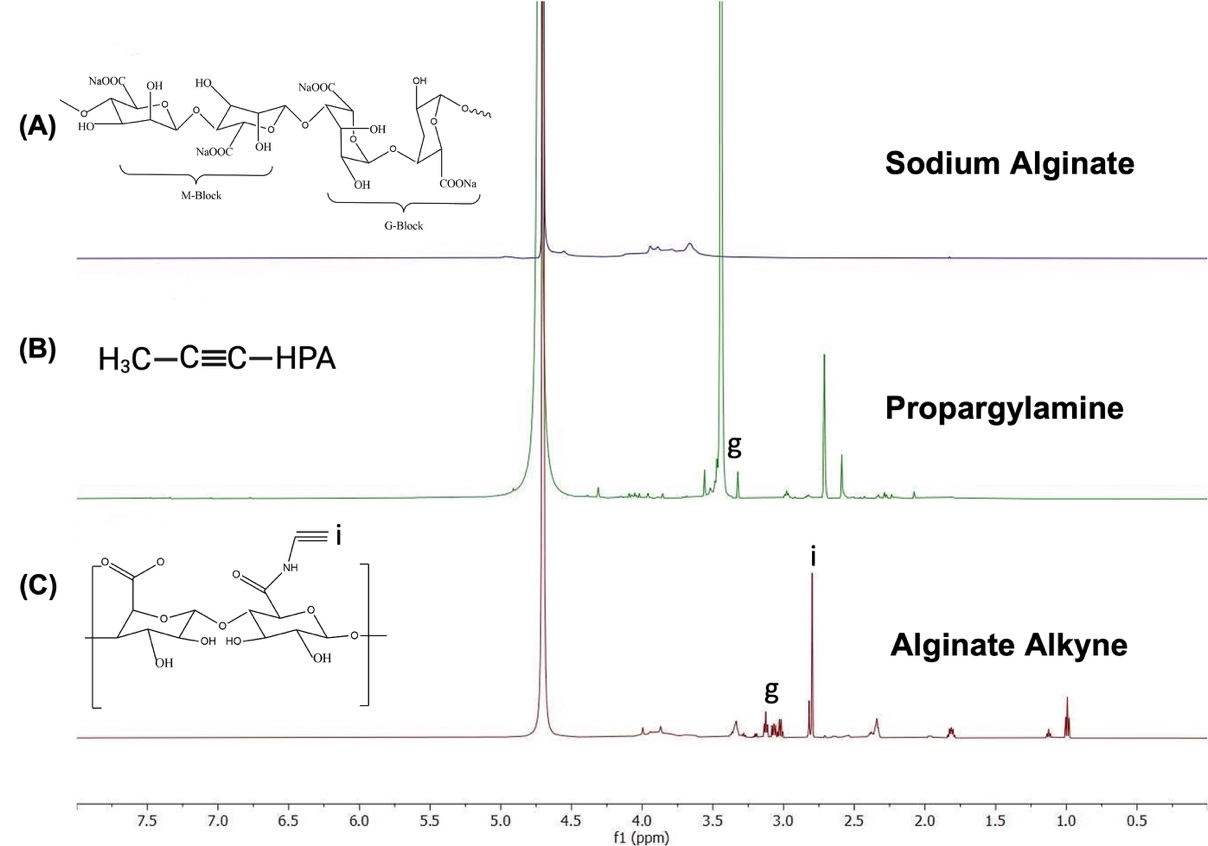


**Figure S5.** The ^1^H NMR spectra confirm the successful conjugation of the alkyne moiety to alginate via amide bond formation. (A) The spectrum of sodium alginate displays its characteristic proton signals. (B) The spectrum of propargylamine peaks at 3.4 ppm (g), corresponding to the propargyl functional group. (C) The spectrum of the alginate-alkyne conjugate reveals the appearance of the alkyne proton peak at 2.8 ppm (i), confirming the incorporation of the alkyne functionality into the alginate backbone. These findings validate the effective conjugation of the alkyne group to the alginate structure.

**
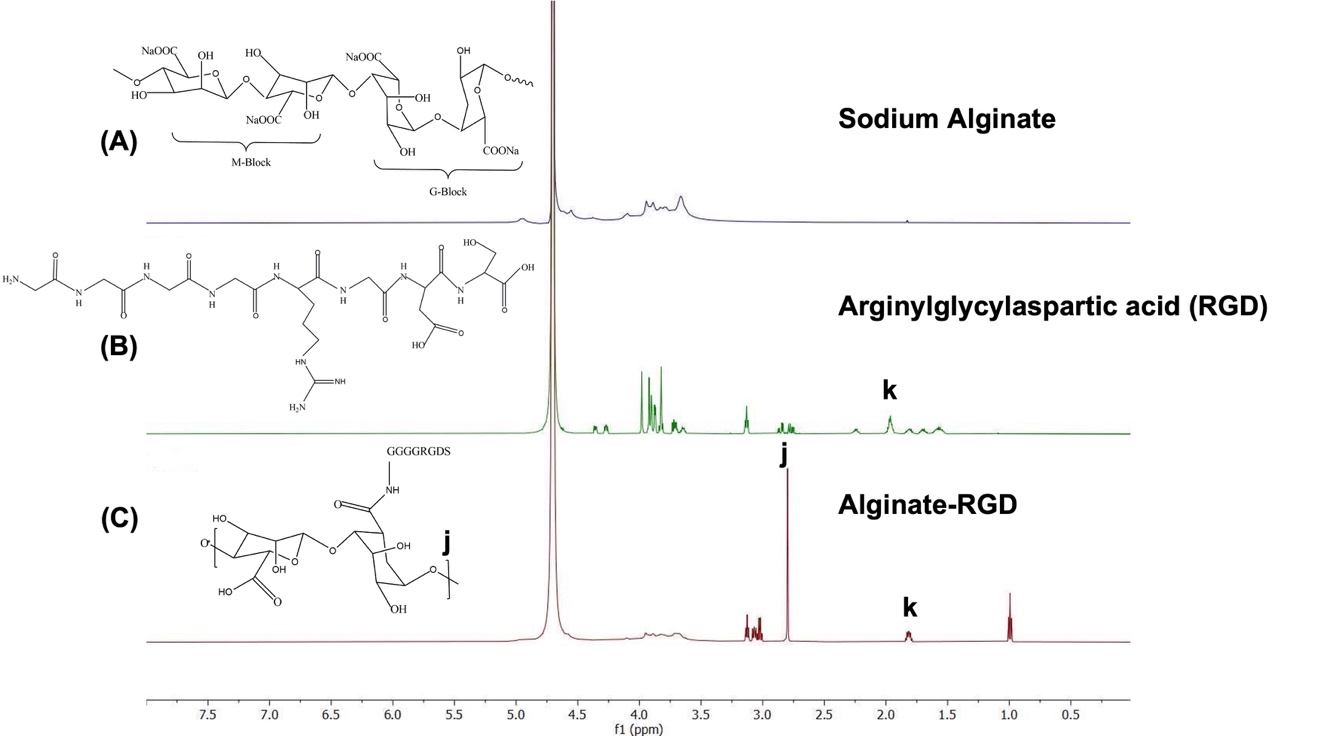
Figure S6.** The ^1^H NMR spectra confirm the successful conjugation of the alkyne moiety via amide bond formation and the incorporation of RGD functionality into the alginate backbone. (A) The spectrum of sodium alginate shows its characteristic proton signals. (B) The spectrum of RGD exhibits a peak at 1.8 ppm (k), corresponding to the proton environment of the RGD functional group. (C) The spectrum of alginate-RGD further supports the successful conjugation, evidenced by the appearance of the alkyne proton peak at 2.7 ppm (j) and the RGD-related peak at 1.7 ppm (k), confirming the co-incorporation of both alkyne and RGD functional groups into the alginate backbone. These results validate the efficient functionalization of alginate with both alkyne and RGD groups.


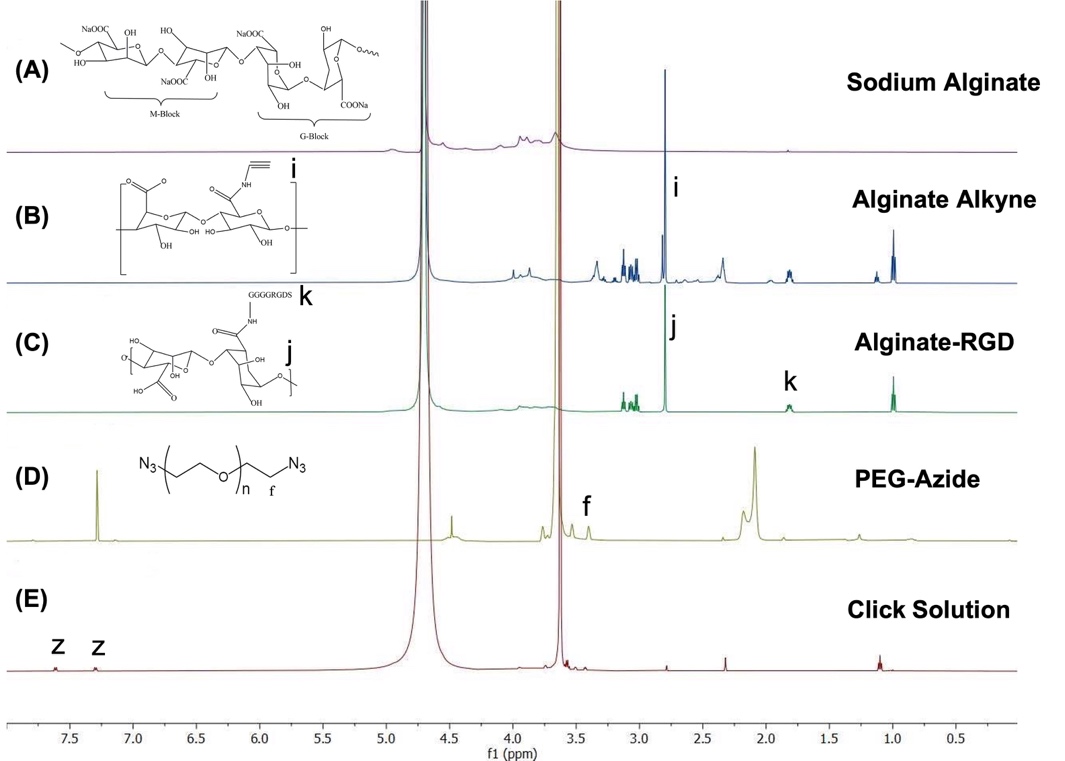


**Figure S7.** The successful functionalization of PEG-alginate-RGD hydrogel via the click reaction was confirmed through ^1^H NMR spectroscopy, as demonstrated by the following observations: (A) Sodium alginate shows its characteristic proton signals; (B) The alkyne signal is observed at 2.7 ppm (i); (C) RGD conjugation is indicated by signals at 1.7 ppm (k) and 2.7 ppm (j); (D) The azide signal appears at 3.33 ppm (f); (E) The presence of triazole rings is confirmed by signals at 7.3 ppm (z) and 7.6 ppm (z).

**3. Stability Test**

**
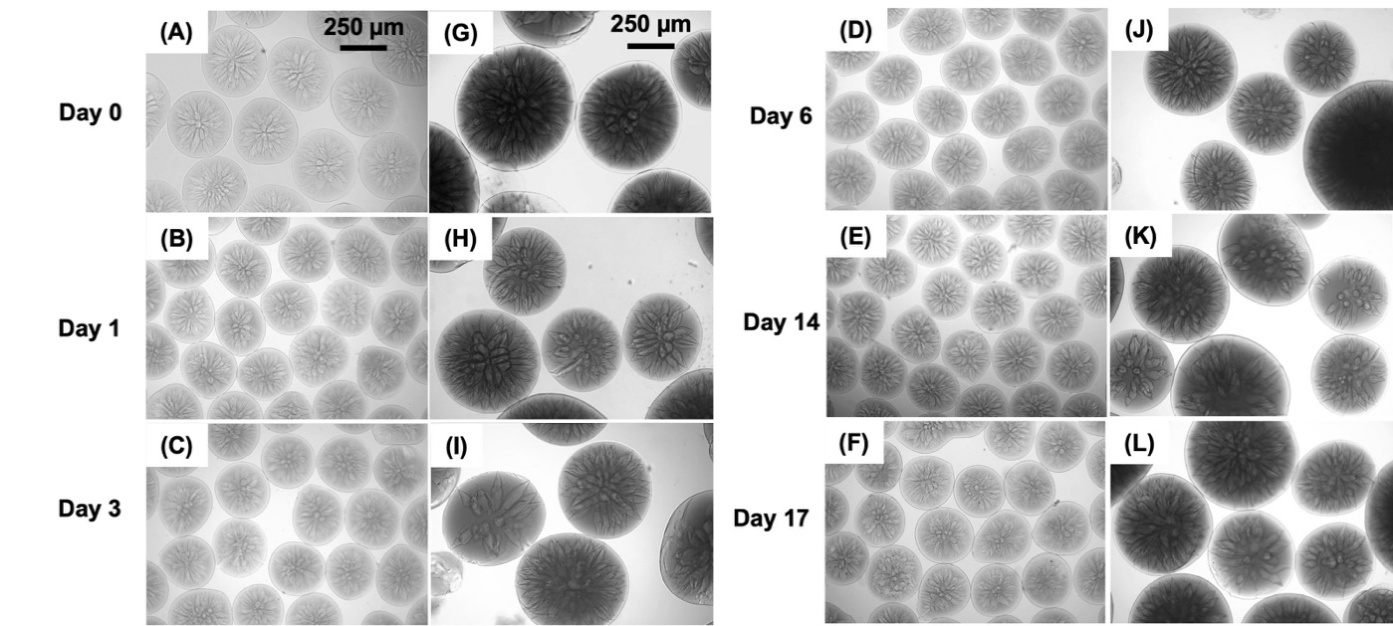
**

**Figure S8.** Stability of 1.5% click microbeads (**A-F**) and 3% click microbeads (**G-L**) in calcium chloride solution over 17 days. The images were captured on days 0, 1, 3, 6, 14, and 17, illustrating changes in the microbead structure over time. (**A, G**) day 0; (**B, H**) day 1; (**C, I**) day 3; (**D, J**) day 6; (**E, K**) day 14; (**F, L**) day 17, all observed at 40× magnification.

**4. Distribution of encapsulated imHCs in Click-RGD microbeads**

**
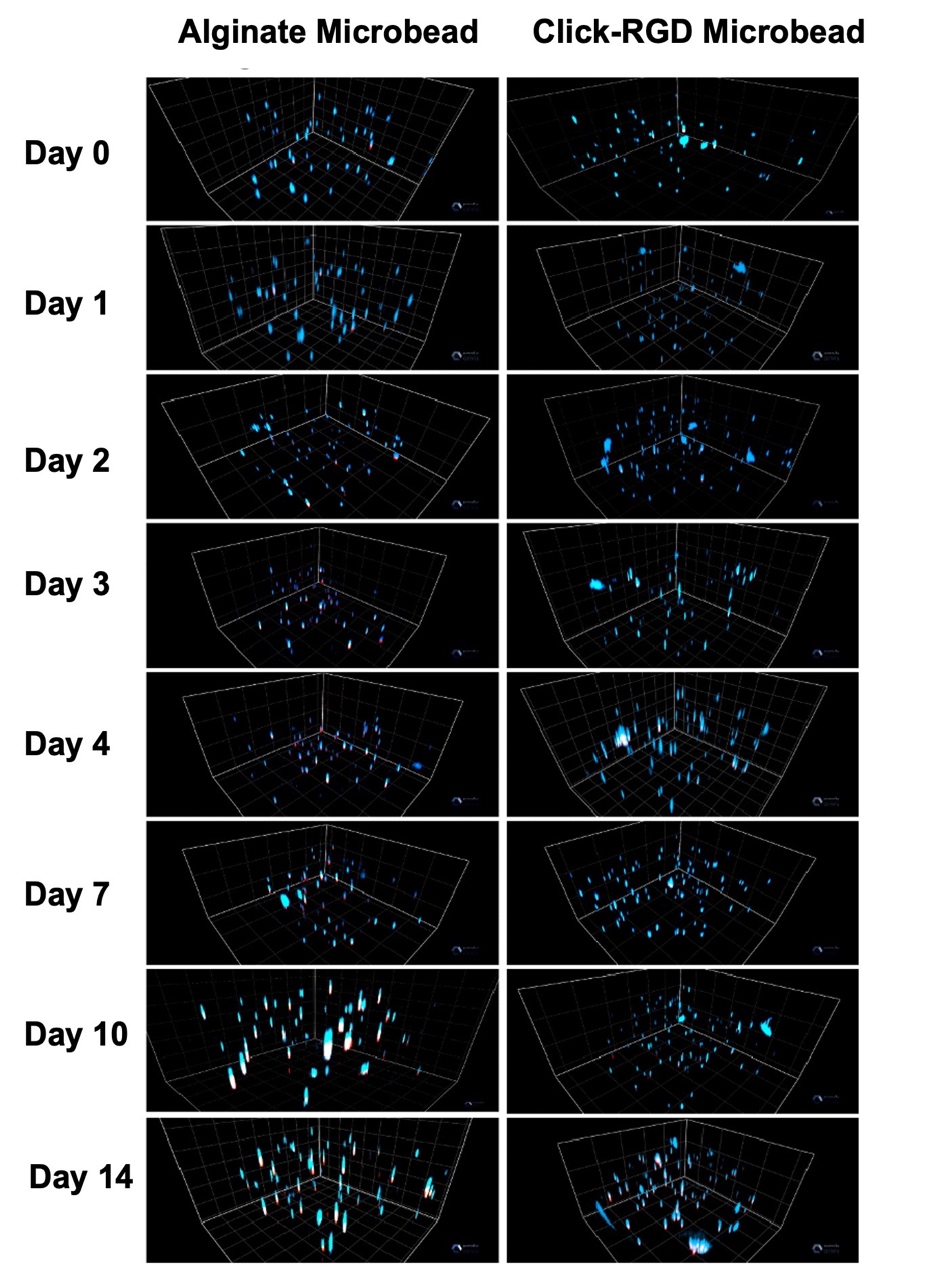
**

**Figure S9.** Distribution of encapsulated imHCs in alginate and click-RGD microbeads. Representative images of imHCs were encapsulated in different microbeads: alginate microbeads (left column) and modified click-RGD microbeads (right column) on days 0, 1, 2, 3, 4, 7, 10, and 14.

**4.** **Pre-injection morphology of imHCs-encapsulated click-RGD microbeads in rats**

The microbeads were functionalized with azide and alkyne groups to facilitate the conjugation of bioactive molecules via click chemistry, allowing for precise control over surface properties and the covalent attachment of RGD peptides, which regulate cellular adhesion, proliferation, differentiation, and anti-apoptotic signaling. (Choe et al., 2019; Wang et al., 2018) RGD conjugation enhanced hepatocyte adhesion via integrin-mediated interactions, supporting cell viability. imHCs (3 × 10⁶ cells/mL) were encapsulated in click-RGD microbeads using the BUCHI Encapsulator B395 Pro, forming microbeads (400-700 µm) with a wrinkled surface, as shown in Figure S10. The optimized formulation improved mechanical strength and structural integrity, making the microbeads suitable for transplantation.

**
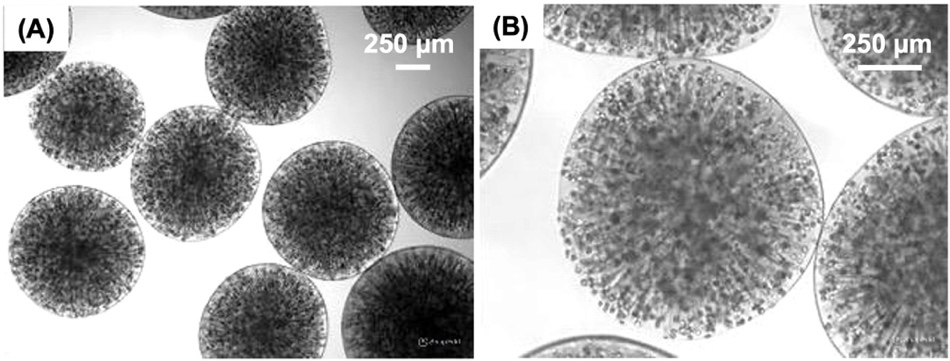
**

**Figure S10.** Bright-field microscopic analysis of imHCs encapsulated in click-RGD microbeads. **(A)** A low-magnification image (40×) illustrates the uniform spherical morphology of the microbeads. **(B)** Higher magnification (100×) reveals a distinct flower-like internal architecture, indicative of successful click conjugation. Encapsulated imHCs appear evenly distributed throughout the microbeads, supporting structural integrity and homogeneous cell incorporation. Scale bars represent 250 μm.

**5. Evaluation of gross anatomy and biocompatibility of CMRL medium and imHCs-encapsulated click-RGD microbeads in a rat model**

A gross anatomical assessment of the peritoneal cavity was performed at the study endpoint, as shown in Figure S11. Rats treated with imHCs-encapsulated click-RGD microbeads displayed normal hepatic morphology, similar to the control group receiving CMRL medium. No inflammation, adhesion, or pathological changes were observed in either group. At the end of the study, the imHC-encapsulated click-RGD microbeads were fully degraded, leaving no residual material in the peritoneal cavity of the experimental rats. These results confirm the biodegradability and biocompatibility of click-RGD microbeads. Furthermore, the absence of inflammatory responses and tissue adhesion supports the potential of this biomaterial for preserving normal liver function without adverse effects, indicating its therapeutic feasibility for supporting liver function during the regeneration process. (Jitraruch et al., 2014) Further histological analysis is needed to confirm biocompatibility in the treated group.


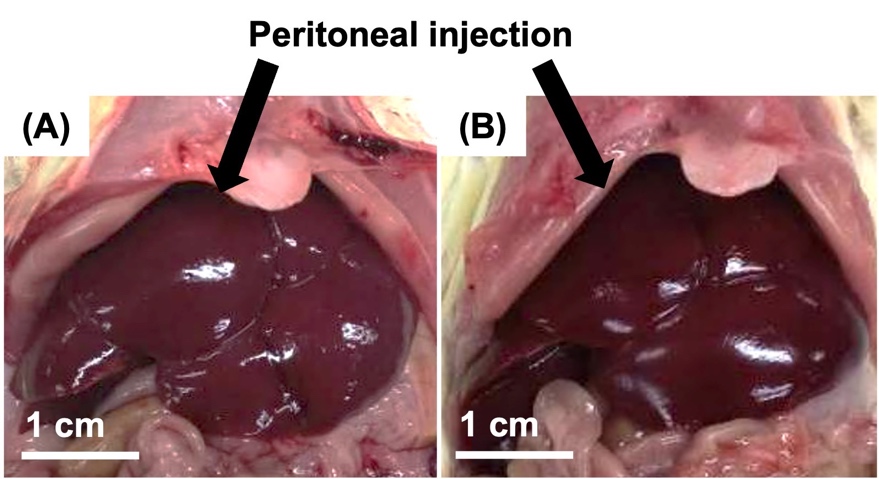


**Figure S11.** Gross examination of the peritoneal cavity of rats at the study endpoint. **(A)** Peritoneal cavity after injection of 3 ml of CMRL medium. **(B)** Peritoneal cavity after injection of imHCs encapsulated click-RGD microbeads in 3 ml of CMRL medium. Both groups showed no signs of inflammation or adhesion.

**6.** **Comparative evaluation of alginate blank microbead and click-RGD blank microbeads for enhanced biocompatibility and degradability in the peritoneal cavity of rats**

The comparison between alginate and click-RGD blank microbeads after injection reveals notable differences in their biocompatibility and degradability (n = 3) on day 14. Click-RGD blank microbeads, enhanced by incorporating RGD peptides, demonstrated superior interaction with the host tissue, facilitating optimal cellular integration and complete degradation through click chemistry. These characteristics make click-RGD blank microbeads a more promising candidate for targeted cell delivery and tissue engineering applications. In contrast, alginate blank microbeads exhibited limited tissue interaction and slower degradation, accumulating away from the liver due to the absence of RGD peptides. This highlights the benefits of click-RGD microbeads in applications that require enhanced biocompatibility and functionality.

**
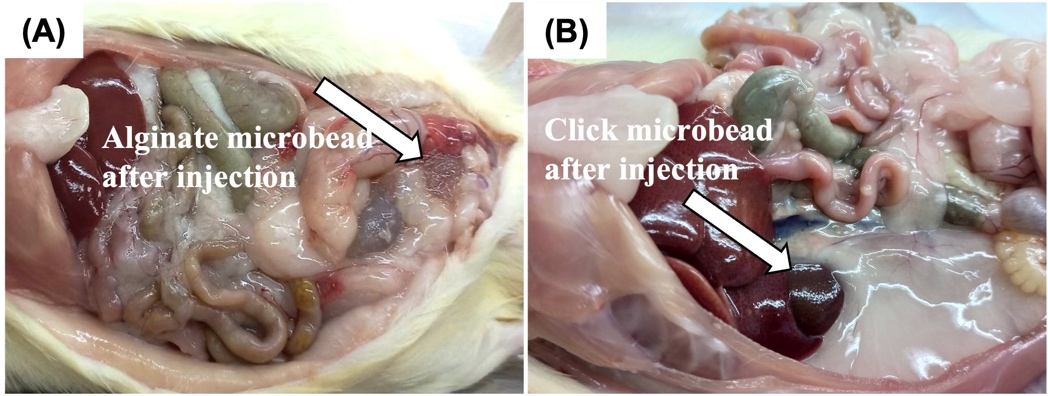
**

**Figure S12.** Comparison of alginate and click-RGD blank microbeads after injection on day 14. **(A)** Alginate blank microbeads after injection and **(B)** Click-RGD blank microbeads after injection into the rat’s peritoneal cavity. The biocompatibility of click-RGD blank microbeads is enhanced due to the presence of RGD peptides, promoting optimal tissue interaction and facilitating complete degradation through click chemistry. Consequently, click-RGD blank microbeads offer targeted cell delivery, improved cellular interaction with the host liver, and superior degradability compared to alginate blank microbeads.

**References**

Chen, J., Liu, Z., Wang, Z., Zhang, X., Zhang, Y., Zhan, Z., Gong, X., & Xu, T. (2024). One-step biofabrication of liquid core-GelMa shell microbeads for in situ hollow cell ball self-assembly. *Regenerative Biomaterials*, *11*, rbae021.

Choe, G., Park, J., Jo, H., Kim, Y. S., Ahn, Y., & Lee, J. Y. (2019). Studies on the effects of microencapsulated human mesenchymal stem cells in RGD-modified alginate on cardiomyocytes under oxidative stress conditions using an *in vitro* biomimetic co-culture system. *International journal of biological macromolecules*, *123*, 512-520.

Edward Semple, J., Sullivan, B., Vojkovsky, T., & Sill, K. N. (2016). Synthesis and facile end‐group quantification of functionalized PEG azides. *Journal of Polymer Science Part A: Polymer Chemistry*, *54*(18), 2888-2895. <https://doi.org/https://doi.org/10.1002/pola.28174>

Jitraruch, S., Dhawan, A., Hughes, R. D., Filippi, C., Soong, D., Philippeos, C., Lehec, S. C., Heaton, N. D., Longhi, M. S., & Mitry, R. R. (2014). Alginate microencapsulated hepatocytes optimised for transplantation in acute liver failure. *PloS one*, *9*(12), e113609. <https://doi.org/https://doi.org/10.1371/journal.pone.0119226>

Patel, S., Patel, S., Kotadiya, A., Patel, S., Shrimali, B., Joshi, N., Patel, T., Trivedi, H., Patel, J., & Joharapurkar, A. (2024). Age-related changes in hematological and biochemical profiles of Wistar rats. *Laboratory Animal Research*, *40*(1), 7.

Rouf, M. A., Das, A. K., Khatoon, M., Mitu, N. L., & Shahriar, M. (2021). Evaluation of hepatoprotective effects of arogyavardhini against D-galactosamine-induced hepatotoxicity in rats. *Journal of Pharmacognosy and Phytochemistry*, *10*(1), 12-19.

Wang, Y.-H., Wu, D.-B., Chen, B., Chen, E.-Q., & Tang, H. (2018). Progress in mesenchymal stem cell–based therapy for acute liver failure. *Stem Cell Research & Therapy*, *9*, 1-9. <https://doi.org/https://doi.org/10.1186/s13287-018-0972-4>

Yang, G., Mahadik, B., Choi, J. Y., Yu, J. R., Mollot, T., Jiang, B., He, X., & Fisher, J. P. (2021). Fabrication of centimeter-sized 3D constructs with patterned endothelial cells through assembly of cell-laden microbeads as a potential bone graft. *Acta Biomaterialia*, *121*, 204-213.
